# Ultrafast Switching of Whispering Gallery Modes in Quantum Dot Superparticles

**DOI:** 10.1021/acs.nanolett.5c00643

**Published:** 2025-02-24

**Authors:** Pietro Castronovo, Marco Reale, Susan A. Rigter, Cherie R. Kagan, Christopher B. Murray, Salvatore Lorenzo, Erik C. Garnett, Peter Schall, Emanuele Marino, Alice Sciortino, Fabrizio Messina

**Affiliations:** † Dipartimento di Fisica e Chimica−Emilio Segrè, 18998 Università degli Studi di Palermo, Via Archirafi 36, 90123 Palermo, Italy; ‡ Center for Nanophotonics, 55952 AMOLF, Science Park 104, 1098XG Amsterdam, The Netherlands; § Van der Waals−Zeeman Institute, University of Amsterdam, Science Park 904, 1098XH Amsterdam, The Netherlands; ∥ Department of Chemistry, 6572 University of Pennsylvania, 231 S. 34th St., Philadelphia, Pennsylvania 19104, United States; ⊥ Department of Materials Science and Engineering, 6572 University of Pennsylvania, 220 S 33rd St., Philadelphia, Pennsylvania 19104, United States; # Department of Electrical and Systems Engineering, 6572 University of Pennsylvania, 200 S. 33rd Street, Philadelphia, Pennsylvania 19104, United States; 7 ATeN Center − Università degli Studi di Palermo, Viale delle Scienze, Edificio 18, 90128 Palermo, Italy

**Keywords:** Nanocrystals, Superparticles, Whispering Gallery Modes, Microresonator, Ultrafast, Transient Absorption Microscopy

## Abstract

Microscopic dielectric structures can leverage geometry and photophysics to confine light, acting as microresonators. However, the use of light to reversibly manipulate the spectral pattern of photonic resonances on ultrafast time scales has hardly been explored. Here, we use femtosecond light pulses to drive reversible changes in the photonic resonances of optical microresonators over a broad spectral range. We employ pump–probe microscopy to investigate the dynamic modulation of the photonic response of whispering-gallery microresonator superparticles self-assembled from colloidal quantum dots. Our findings provide crucial insight into the photophysics of semiconductor superstructures, paving the way to their prospective application as ultrafast optical switches for photonics, optoelectronics, and communication technologies. In particular, we demonstrate that ultrafast photoexcitation can initiate ultrafast excitation transfer between neighboring superparticles, forming a dimer, and induce electronically and thermally driven changes in the refractive index of individual superparticles, dynamically modulating their resonances on distinctive time scales.

## Graphical abstract

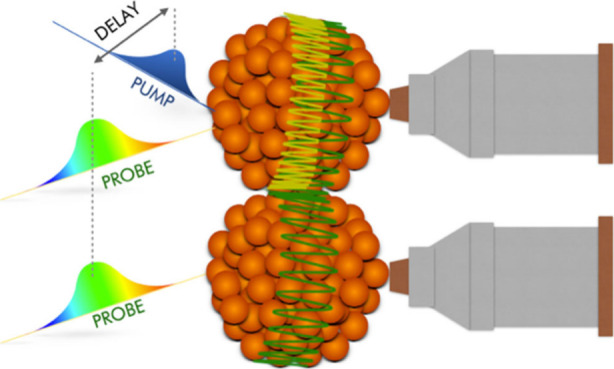

The organized assembly of nanocrystals into mesoscopic structures introduces new properties stemming from interactions between constituents.^1,2^ The observation of these effects has been facilitated by recent advancements in synthetic methods enabling the synthesis of near-monodisperse nanocrystals and their self-organization into controlled three-dimensional structures with crystalline, quasi-crystalline, or amorphous organization, denominated superparticles (SPs).^3,4^ Notable examples include perovskite supercubes,^5,6^ quantum dot (QD) superspheres,^7,8^ faceted plasmonic nanoparticle supercrystals,^9,10^ and hollow metal nanocluster superspheres.^11^ Indeed, any type of nanoparticle, such as metal chalcogenide^7^ or perovskite^5^ QDs and nanorods,^12^ nanometals,^9^ magnetite nanocubes,^13^ and branched colloidal nanocrystals,^14^ can serve as functional building blocks of artificial solids displaying novel properties like exciton delocalization and band-like transport,^15–18^ collective plasmonic responses,^9,19,20^ ultraefficient surface-enhanced Raman scattering,^21,22^ superfluorescence,^5,23,24^ long-range charge or energy transport,^25–28^ photonic and excitonic coupling,^17,18,29^ or aggregation-induced photoluminescence.^11^ Notably, circularly symmetric SPs can support whispering gallery modes (WGMs), sharp optical resonances due to quasi-total internal reflection of light within a round dielectric cavity, strongly influenced by the SP’s radius and refractive index.^30–32^ SPs whose light absorption and/or emission spectrally matches the resonator modes behave as active microresonators, a novel class of systems showing great promise for applications such as fine-tunable microlasers^33–36^ and anticounterfeiting microlabels.^37^ Although these artificial solids have the potential to result in real-world applications in photonics and optoelectronics, their fundamental physical understanding remains at an early stage. Besides, existing studies have scarcely addressed how light can induce ultrafast reversible changes in the wide-band optical response of SPs, a concept that may enable a wide range of new applications in photonics.

Addressing the functional optical response of SPs requires experimental methods capable of spatial resolution comparable to the SP size (μm) and of resolving the dynamics initiated by photon absorption down to the pico- and femtosecond time scale. These are both the typical time scale of semiconductor QD relaxations^38,39^ and of relevant cross-talk and collective phenomena involving the whole SP, such as long-range excitation migration or light propagation over micrometer distances. Indeed, previous studies^40,41^ on the optical modulation of microresonators were typically limited in temporal and spectral resolution.

To meet such demands for high spatial and temporal resolution, we employed ultrafast pump–probe microscopy (μPP),^42,43^ a recently developed technique able to provide unique insight into the photophysics of SPs. Indeed, in a typical pump–probe transient absorption experiment, the sample is excited by a femtosecond pulsed optical beam (pump), and its photoinduced differential absorption is measured by another delayed pulsed beam (probe), whereas μPP augments transient absorption measurements by adding optical microscopy.^44^ We achieve μPP by tightly focusing the pump and probe pulses on the sample and collecting the transmitted probe through a microscope objective, gaining spatially resolved insight into the ultrafast photodynamics. Recently, μPP was used to address nanostructured systems such as perovskite^45,46^ and metal chalcogenide QD^47,48^ films and organic–inorganic heterostructures.^49^ However, broadband, femtosecond-resolved μPP has not yet been used to probe the photoinduced changes in optical microresonators, despite its potential to provide key insight into their optical response and functional photonic behavior.

Figure 1a illustrates the concept of this work. We study quasi-monodisperse spherical SPs, assembled from semiconductor QDs via a synthetic approach recently demonstrated by some of the authors.^7^ These SPs behave as active microresonators with the QD superstructure serving as both active medium and resonator cavity.^34^ We use μPP to photoexcite and interrogate individual SPs, allowing to track WGM modulation effects with simultaneously high temporal (≃50 fs), spectral (≃0.5 nm), and spatial (≃2.0 μm) resolutions (Paragraph 1.7 of the SI, Figure S3). Thus, we fully reconstruct how the whole spectral pattern of the microresonator dynamically evolves on ultrafast time scales and gain key insight into the optical response and functional photonic behavior of the SPs. Our approach allows to unravel the fundamental electronic and thermal effects behind the photoinduced dynamic variations of SP resonator modes, which may allow using SPs as ultrafast, reversible light-driven microswitches. We also observe in real-time the ultrafast energy transfer between neighboring SPs forming a dimer, opening exciting perspectives to applications of SPs at a higher hierarchical degree of assembly.

**Figure 1:**
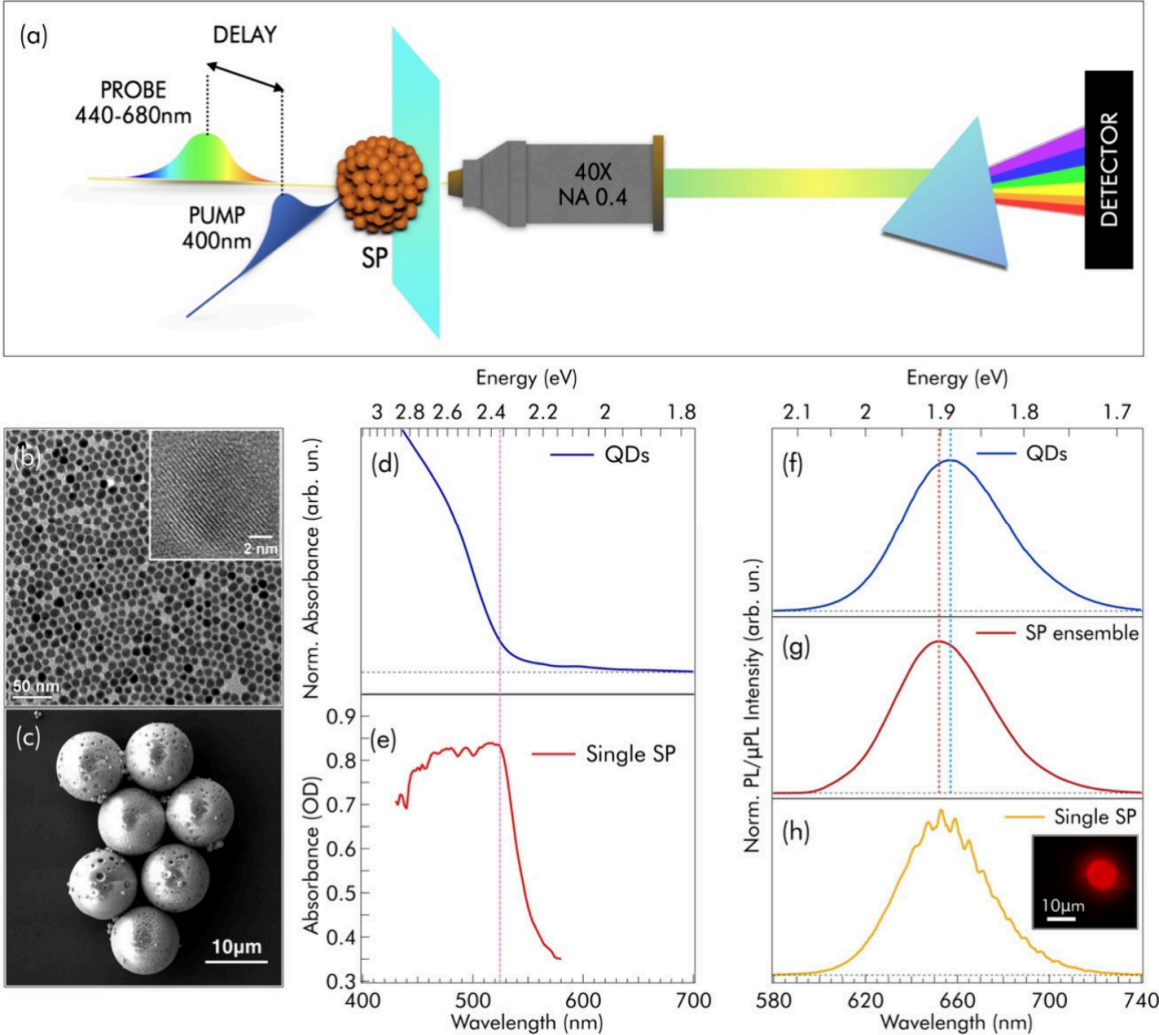
(a) Schematic representation of μPP measurements performed on individual QD SPs. A single SP is excited by pulses with 50 fs duration, a wavelength of either 400 or 550 nm, and a spot size ranging from 2.0 to 15 μm. The excited SP is then probed by a tightly focused white-light femtosecond probe beam (2.0–2.5 μm fwhm diameter) aligned at the center and subsequently collected via a microscope objective. (b) TEM image of the CdSe/CdS core/shell QDs. The inset shows an HRTEM image of a single QD. (c) SEM image of a group of QD SPs, highlighting their accurate spherical shape and very low polydispersity. (d) UV–vis spectrum of a colloidal dispersion of QDs. (e) μ-UV–vis absorption spectrum of a single SP obtained via integrating sphere microscopy, as described in the SI (Paragraph 1.4) and ref 50. (f) Steady-state photoluminescence of a colloidal dispersion of QDs. (g) Steady-state photoluminescence of QD SPs at the ensemble level, where the vertical dotted lines highlight the shift with respect to the QDs’ emission. (h) μPL spectrum of a single SP, displaying whispering gallery mode resonances. The inset shows a fluorescence micrograph of an individual SP.

Figure 1b shows a TEM micrograph of the oleate-capped, 10.3 nm CdSe/CdS core/shell QDs (core diameter 3.5 nm) constituting the SPs, while Figure 1c reports a typical SEM image of the highly monodispersed SPs, 10.8 ± 0.1 μm in diameter, each containing more than 10^8^ QDs. Figure 1 compares steady-state optical measurements on the QDs (Figures 1d and 1f) and their SPs (Figures 1e, 1g, and 1h). The QDs (Figure 1d) strongly absorb light with wavelengths λ < 520 nm due to their thick CdS shell (bandgap = 2.42 eV = 512 nm), while the much weaker absorption at λ > 520 nm stems from the quantum-confined CdSe core. The assembly of QDs into SPs significantly increases light scattering, making a traditional absorption measurement impossible. Thus, we used an integrating sphere-based microabsorption method^50^ to remove the scattering contribution from the extinction spectrum of a single SP. Figure 1e displays one such measurement (see Figure S1 for additional spectra). The absorption cross-section of SPs shows saturation at short wavelengths (λ < 520 nm), a behavior already observed by some of the authors.^17^ This was previously attributed to the strong photonic coupling between QDs allowing the SP to reach an absorption cross-section σ comparable to the geometrical cross-section π*a* ^2^, in contrast to individual QDs, for which σ ≪ π*a* ^2^.

Thanks to the strongly confined CdSe core, both QDs and SPs are highly photoluminescent, with comparable quantum yields of ≃90%. Ensemble measurements of well-dispersed QDs and SPs show smooth emission spectra differentiated by a 5 nm shift (Figures 1f and 1g). Microphotoluminescence measurements of individual SPs reveal their light-coupling properties (Figure 1h and Figure S2). Regular, narrow peaks appear on top of the broad fluorescence envelope due to the microresonator SP selectively enhancing emission rates at its resonance frequencies.^51^ The modulations appear more intense on the red side of the envelope, likely because of increasing optical losses on the blue side.^33,52^ A direct comparison of several SPs (Figure S2b) shows that the position and relative intensities of the modulations are unique to each SP, owing to the strong dependence of WGMs on the size and shape variations among SPs. This causes the WGM features (Figure 1h) to average out in an ensemble spectrum (Figure 1g). WGMs should also appear in microabsorption spectra (Figure 1e). However, these measurements mainly cover the region of high light absorption, where the quality factor of WGMs is low and the instrument’s spectral resolution (3 nm) is insufficient.

In light of the intriguing steady-state properties of our SPs, we proceeded to use μPP to explore their time-dependent excitation and relaxation. Contrary to traditional pump–probe spectroscopy, μPP allows isolating genuine absorption variations from scattering contributions (as discussed in Paragraph 1.9 of the SI) and revealing photoinduced WGMs changes by probing single SPs rather than an ensemble.

Figure 2 summarizes the typical relaxation behavior of a single SP, as revealed by μPP measurements. The SP, deposited on a glass substrate, was excited with 0.3 mJ/cm^2^, 50 fs light pulses at 400 nm and probed in the range 440–680 nm at several time delays from photoexcitation. In this case we employed a 14.0 μm FHWM pump to ensure that the SP is uniformly illuminated, and a much smaller (<2.5 μm FHWM) probe, aligned at the center of the SP. Differential μPP absorption spectra at varying pump–probe delays are displayed in Figure 2a as a heat map. From this map, we extract spectra at specific delays (Figure 2b) and kinetic traces (Figure 2c) by vertical and horizontal cuts, respectively. The inset in Figure 2b shows that the μPP spectra display regular modulations over the broad signal envelope. This intriguing aspect will be thoroughly discussed after analyzing the underlying broader spectral shape.

**Figure 2:**
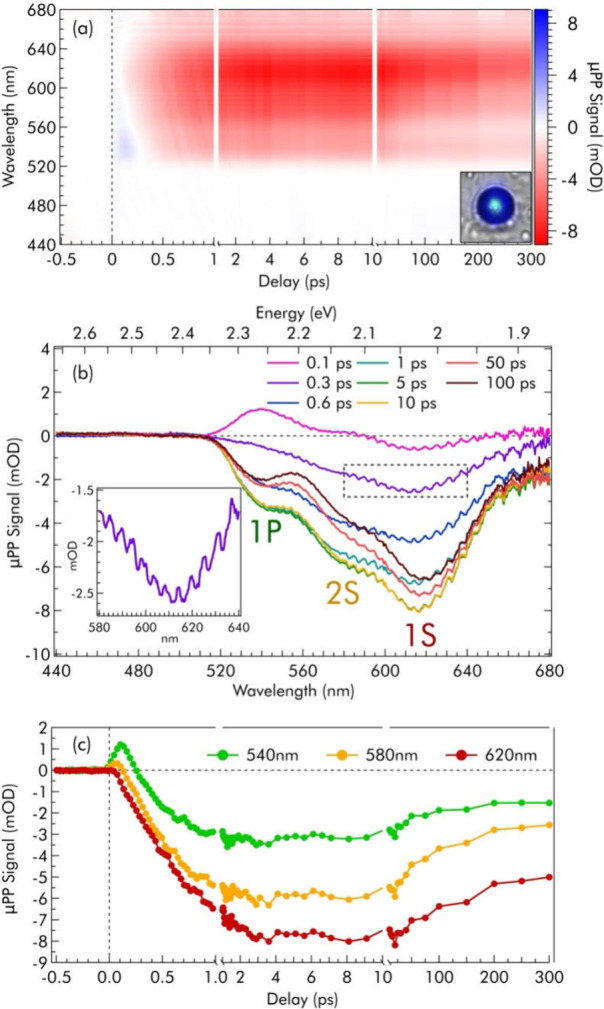
μPP measurements on a single QD superparticle (SP). (a) Two-dimensional time-wavelength plot of the pump–probe signal of the single SP shown in the inset and (b) μPP spectra at fixed pump–probe delays extracted from vertical cuts of panel (a). The labels 1S, 2S, and 1P indicate GSB signals corresponding to known transitions of CdSe QDs (see details in the text), while the inset highlights the presence of modulations over the envelope of the pump–probe signal. (c) Kinetic traces extracted as horizontal cuts of panel (a) at fixed wavelengths.

So-called state-filling effects^53^ dominate the photoinduced dynamics of our SPs, leading to ground state bleaching (GSB) features appearing at the position of allowed optical transitions. Indeed, the μPP spectra of Figure 2b display GSB peaks at ≃615 nm (2.02 eV), ≃580 nm (2.16 eV), and ≃545 nm (2.28 eV), coherently with known excitonic transitions for CdSe QDs,^53^ namely 1S [1S­(e)–1S_3/2_(h)], 2S [1S­(e)–2S_3/2_(h)], and 1P [1P­(e)–1P_3/2_(h)]. Interestingly, the overall shape of this μPP signal differs significantly from that of a drop-casted colloidal solution of QDs (Figure S5), where the GSB signals are shifted and have different intensity ratios. The signals rise in ≃3 ps, because of the relaxation of charge carriers from highly excited states down to the band edges driven by electron–phonon scattering. We also observe a short-lived photoinduced absorption around 540 nm (2.30 eV). The whole signal partially decays on a time scale of hundreds of picoseconds due to radiative and nonradiative depopulation of the lowest excited state. Furthermore, the signal at λ < 520 nm is zero at all delays (Figure 2c), despite probe light in that spectral range still partially penetrating the SP (Figure S6). This expands on the saturation effect observed in micro-OA (Figure 1e), highlighting that the saturated absorption cross-section is unaffected by photoexcitation.

We explored the variability among different SPs by comparing their μPP signals obtained under identical experimental conditions (0.3 mJ/cm^2^ pump pulses at 400 nm). While the main μPP spectral features are always present, their intensity ratios and exact spectral positions differ. For example (Figure 3b), the lowest-energy 1S bleach peak at short delays is located at 614, 604, and 606 nm for the three SPs. This result emphasizes the need to study the SPs individually, as their absorption fingerprint is a unique and collective property of each SP, conditioned by minute changes in their size (1% polydispersity), shape, and refractive index. Despite these differences, the kinetic traces extracted at the GSB positions are identical (Figure S10), suggesting a common relaxation pathway following photoexcitation.

**Figure 3:**
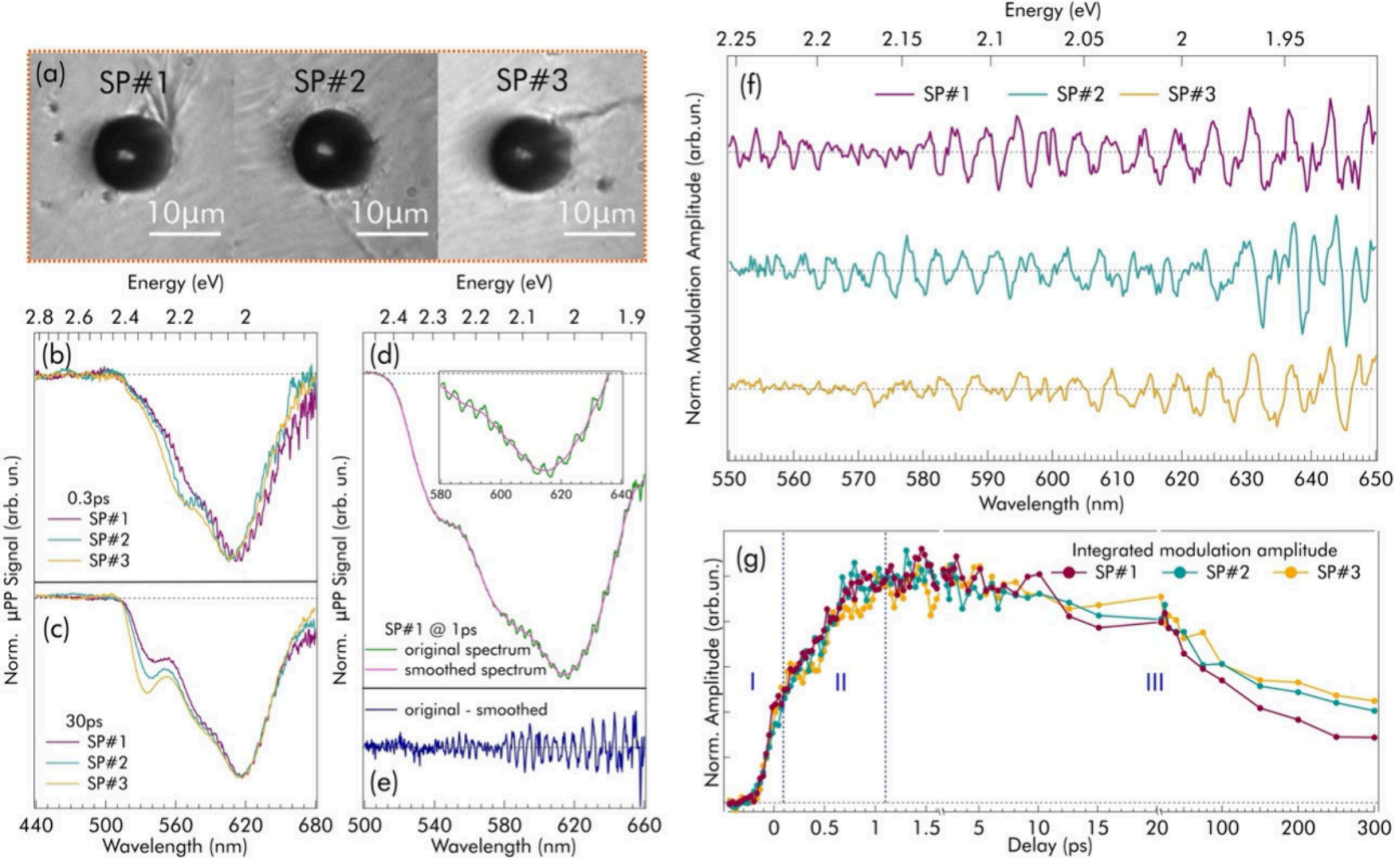
Unraveling the photonic properties of single SPs via μPP: (a) optical microscopy images of three distinct SPs (SP#1, SP#2, and SP#3) on the same substrate; (b and c) normalized μPP spectra of SP#1, SP#2, and SP#3 at 0.3 ps (b) and 30 ps (c) delays, highlighting the different modulations and their time evolution; (d and e) exemplification of the analysis procedure used to extract the modulations; (f) modulations of SP#1, SP#2, and SP#3 at 1 ps delay, extracted as shown in (d) and (e); and (g) time evolution of the overall modulation amplitude for all three SPs. The vertical dashed lines separate the three phases of the dynamics, as indicated by the labels I–II–III.

We now focus on the most peculiar feature of these μPP signals: a complex modulation structure appearing over the broad signal envelope (inset of Figure 2b and Figures S7 and S9). To study the nature of the modulations, we isolate them from the signal envelope by subtracting, at each pump–probe delay, a smoothed version of the signal (Figures 3d and 3e), obtaining the residual signals shown in Figure 3f.

The coarse structure of these spectral modulations is a progression of negative and positive lobes in close succession. Considering the nature of our SPs as microresonators, we link these modulations with the microresonator modes, known to be extremely sensitive to the refractive index and radius of the resonator.^54^ This link is more subtle than it seems: because of the differential nature of the μPP technique, any signal results from a change between the photoexcited and the nonexcited state of the system. Therefore, we conclude that these transient modulations arise from a modification of the WGM resonances, which can only be due to a photoinduced change Δ­(*an*) of the refractive index *n* and/or the radius *a* of the SP. Thus, μPP is successful in interrogating and probing the photoinduced changes in the frequency structure of the microresonator.

Several observations corroborate our interpretation. First, the precise spectral positions and amplitudes of these modulations vary between SPs (Figure S8), consistently with the changes in WGM progression observed in the microphotoluminescence spectra (Figure S2b). Second, the modulations observed in μPP become less intense in the blue and disappear entirely below ≃530 nm, coherently with the decreasing quality factor of WGM resonances due to increasing optical losses (Paragraph 2.1 of the SI). Third and most important, if WGMs are altered by photoexcitation, we should observe negative (bleach) and positive peaks in the differential signal, located spectrally close to the original and shifted positions of the WGMs, respectively. Figure S11 shows a linear fit of the progression of negative peaks from Figure 3e. Consistently with our interpretations, the result matches very well with the first-approximation theoretical expression for the frequencies of WGMs of a spherical resonator ( $\nu_{l}≃c\frac{l}{2\pi an}$ , where *c* is the speed of light, *a* is the average radius of our SPs, and *l* is an integer mode index), allowing the estimation of the real part of the average refractive index as *n*_avg_ = 2.19 ± 0.02 (Paragraph 2.2 of the SI).

As detailed in Paragraph 2.3 of the SI, from the average distance between a given negative peak and its nearest positive counterpart shown in Figure 3e, we can estimate that the quantity *an* varies by (0.34 ± 0.13)%. If we assume that no radius variation Δ*a* occurs, then this corresponds to a photoinduced refractive index variation of Δ*n* = 0.007 ± 0.002.

To obtain further insight into the origin of these effects, we correlate the time dependence of the modulations to the known dynamics of QD relaxation.^53^ The spectrally integrated absolute value of the modulation amplitude *A*_m_ (λ), given by ∫_λ_in__^λ_fin_^|*A*_m_(λ)|dλ (where λ_in_ and λ_fin_ indicate the limits of the probed range), is plotted in Figure 3g and Figure S12 as a function of delay. This shows that the modulations appear within the cross-correlation time of the excitation pulse (phase I), already rising to ≃40% of their maximum amplitude within 0.15 ps. The modulations rise further on a time scale of ≃1 ps (phase II), concurrently with state filling dynamics (Figure 2c), and slowly decay (phase III) over hundreds of picoseconds, although faster than the overall μPP signal (Figure S12). The quasi-instantaneous appearance of the modulations in phase I, that is, much faster than electron–phonon scattering,^53^ strongly hints at a purely electronic effect, namely the variation of the refractive index Δ*n*_el_ caused by the impulsive redistribution of electrons upon photoexcitation. Indeed, no change in radius Δ*a* could occur before electrons have transferred energy to the lattice. Conversely, we propose that the further rise of the modulations upon electron–phonon scattering (phase II) is thermal in origin, as the macroscopic SP temperature rise, through redistribution of energy from electrons to phonons, is expected to induce an additional contribution Δ*n*_th_ and possibly also a change of the radius Δ*a*_th_ due to the thermal expansion of the SP.

Following these considerations and the short penetration of 400 nm photons within the SP (≃100 nm), we calculate the near-surface temperature rise upon photoexcitation to be ≃7K (see SI Paragraph 2.4 for calculation details) and the resulting Δ*n*_th_ to be ≃0.003. This sizeable portion of the overall variation calculated above confirms its partial thermal origin. Moreover, because the combination of Δ*n*_el_ and Δ*n*_th_ accounts for most of the estimated total variation Δ*(an)*, we infer that the effects of Δ*a*_th_ are minor, as also confirmed by a order-of-magnitude estimate based on known thermal expansion coefficients (SI Paragraph 2.4).

These results confirm our attribution of the modulations observed in the μPP signal to photoinduced modification of the WGMs of the SP microresonator. Photoexcitation acts as an all-optical switch^55^ capable of modulating the microresonator on ultrafast time scales, conceptually akin to an acousto-optic modulator, or a variable impedance element in a radiofrequency device. Alternatively, we can picture the photoexcited SP as an ultrafast photonic device, encoding its unique spectral signature into the probe beam, ultimately transmitting this spectral “key” to the detector. Once scalability and other practical issues are overcome, this paradigm might lead to applications in various fields, such as remote recognition, ultrafast telecommunication, or information processing.

Notably, the μPP signal obtained by exciting an SP at 550 nm does not show modulations (Figure S14). While, at 400 nm, the penetration depth (≃100 nm) is comparable to the radial extent of WGMs, at 550 nm, the whole SP is photoexcited. Because the photoexcited QDs are now distributed over the entire volume rather than in the volume probed by the WGMs, we expect both purely electronic and thermal effects to decrease substantially when exciting at 550 nm.

As a final step of our experiment, we push the capabilities of μPP to explore the possibility of cross-talk between neighboring SPs. The deposition of SPs on a glass substrate causes the formation of multimers following Poissonian statistics (Figure S15).^7^ We expect these multimers to support light propagation only through resonator modes that are common to all neighboring SPs. We explore this possibility in a SP dimer by using μPP. First, we focus the pump beam tightly (3 μm fwhm, 4 mJ/cm^2^) to excite only one of the two SPs (site I in Figure 4a). Then, we align the probe with the center of the other SP (site II). This configuration monitors exclusively the transfer of optical excitation through the dimer from one SP to the other. We perform a control experiment on the same SP dimer by exciting and probing site II (Figure 4b). The results shown in Figure 4c confirm the occurrence of excitation transfer: following the photoexcitation of the first SP, we observe a signal from the second SP. Interestingly, this signal is delayed by τ_D_ = 140 fs with respect to the case of direct excitation (Figure 4b and the associated fit in Figure S16). This delay is consistent with the propagation of light in a medium with *n*_avg_ = 2.19 across a distance of about 21 μm. Importantly, this propagation length is about twice as large as the 10.8 μm center-to-center distance between the SPs (corresponding to the distance between pump and probe spots), suggesting that light propagates via the perimeter of the SPs through WGMs. Excitation transfer is further confirmed in images of the scattered pump (inset of Figure 4c), showing that a small (2.5%) portion of the pump is transferred from the initially excited SP to its neighbor. A further extension of these studies to more complex multimer geometries may establish the grounds to use SPs as fundamental building blocks of all-optical circuits.

**Figure 4:**
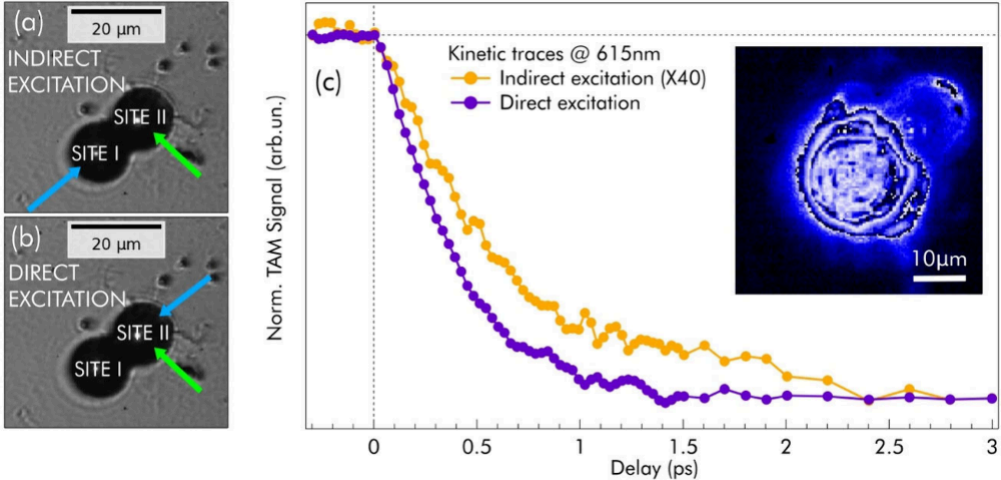
Unraveling the photonic coupling in SP dimers. (a and b) Optical micrograph of a SP dimer: the SPs are labeled “site I” and “site II”, while the blue and green arrows show the position of the pump and probe, respectively, in the case of indirect (a) and direct (b) pumping. (c) Kinetic traces of the μPP signal at 615 nm in the indirect (orange curve, multiplied by 40) and direct (purple curve) pumping regimes. The inset shows a false-color micrograph of the scattered pump beam, highlighting the partial excitation transfer from site I to site II. The two kinetics are normalized to the same intensity at a delay of 3.0 ps.

In summary, we demonstrated that ultrashort light pulses can drive reversible changes in the microresonator response of QD superparticles and that the combined temporal and spatial resolution of μPP can provide key information on the fundamental underlying mechanisms, disentangling electronic and thermal effects. Furthermore, our experiment allows one to directly track in real-time the ultrafast excitation transfer through common WGMs within an SP dimer. While still far from practical implementation, these findings provide the foundation for a future application of QD SPs as optical metamaterials for photonics and optoelectronics. A more comprehensive theory will be needed to predict new photonic effects to target experimentally and expedite future technological developments.

## Supplementary Material



## Notes

Details on the synthesis and structural and optical characterization; experimental methods, theoretical simulations, and supplementary discussions; and auxiliary figures (PDF)

## References

[1] Boles M. A., Engel M., Talapin D. V. (2016). Self-Assembly
of Colloidal Nanocrystals:
From Intricate Structures to Functional Materials. Chemical Reviews.

[2] Murray C. B., Kagan C. R., Bawendi M. G. (2000). Synthesis and Characterization
of
Monodisperse Nanocrystals and Close-Packed Nanocrystal Assemblies. Annu. Rev. Mater. Sci..

[3] Ye X., Chen J., Eric Irrgang M., Engel M., Dong A., Glotzer S. C., Murray C. B. (2017). Quasicrystalline
Nanocrystal Superlattice
with Partial Matching Rules. Nat. Mater..

[4] de
Nijs B., Dussi S., Smallenburg F., Meeldijk J. D., Groenendijk D. J., Filion L., Imhof A., van Blaaderen A., Dijkstra M. (2015). Entropy-Driven Formation of Large Icosahedral Colloidal
Clusters by Spherical Confinement. Nat. Mater..

[5] Rainò G., Becker M. A., Bodnarchuk M. I., Mahrt R. F., Kovalenko M. V., Stöferle T. (2018). Superfluorescence from Lead Halide Perovskite Quantum
Dot Superlattices. Nature.

[6] Tang Y., Gomez L., Lesage A., Marino E., Kodger T. E., Meijer J.-M., Kolpakov P., Meng J., Zheng K., Gregorkiewicz T., Schall P. (2020). Highly Stable Perovskite Supercrystals
via Oil-in-Oil Templating. Nano Lett..

[7] Marino E., van Dongen S. W., Neuhaus S. J., Li W., Keller A. W., Kagan C. R., Kodger T. E., Murray C. B. (2022). Monodisperse Nanocrystal
Superparticles through a Source–Sink Emulsion System. Chem. Mater..

[8] Marino E., LaCour R. A., Kodger T. E. (2024). Emergent
Properties from Three-Dimensional
Assemblies of (Nano)­Particles in Confined Spaces. Cryst. Growth Des.

[9] Kwon N., Oh H., Kim R., Sinha A., Kim J., Shin J., Chon J. W. M., Lim B. (2018). Direct Chemical Synthesis of Plasmonic
Black Colloidal Gold Superparticles with Broadband Absorption Properties. Nano Lett..

[10] Kim S., Zheng C. Y., Schatz G. C., Aydin K., Kim K.-H., Mirkin C. A. (2020). Mie-Resonant Three-Dimensional
Metacrystals. Nano Lett..

[11] Jash M., Jana A., Poonia A. K., Khatun E., Chakraborty P., Nagar A., Ahuja T., Adarsh K. V., Pradeep T. (2023). Phosphine-Protected
Atomically Precise Silver–Gold Alloy Nanoclusters and Their
Luminescent Superstructures. Chem. Mater..

[12] Wang T., Zhuang J., Lynch J., Chen O., Wang Z., Wang X., LaMontagne D., Wu H., Wang Z., Cao Y. C. (2012). Self-Assembled Colloidal Superparticles from Nanorods. Science (1979).

[13] Singh G., Chan H., Baskin A., Gelman E., Repnin N., Král P., Klajn R. (2014). Self-Assembly of Magnetite Nanocubes
into Helical Superstructures. Science (1979).

[14] Miszta K., de Graaf J., Bertoni G., Dorfs D., Brescia R., Marras S., Ceseracciu L., Cingolani R., van Roij R., Dijkstra M., Manna L. (2011). Hierarchical Self-Assembly
of Suspended Branched Colloidal Nanocrystals into Superlattice Structures. Nat. Mater..

[15] Lan X., Chen M., Hudson M. H., Kamysbayev V., Wang Y., Guyot-Sionnest P., Talapin D. V. (2020). Quantum Dot Solids
Showing State-Resolved Band-like Transport. Nat. Mater..

[16] Choi J.-H., Fafarman A. T., Oh S. J., Ko D.-K., Kim D. K., Diroll B. T., Muramoto S., Gillen J. G., Murray C. B., Kagan C. R. (2012). Bandlike Transport in Strongly Coupled
and Doped Quantum
Dot Solids: A Route to High-Performance Thin-Film Electronics. Nano Lett..

[17] Marino E., Sciortino A., Berkhout A., MacArthur K. E., Heggen M., Gregorkiewicz T., Kodger T. E., Capretti A., Murray C. B., Koenderink A. F., Messina F., Schall P. (2020). Simultaneous
Photonic and Excitonic Coupling in Spherical Quantum Dot Supercrystals. ACS Nano.

[18] Crisp R. W., Schrauben J. N., Beard M. C., Luther J. M., Johnson J. C. (2013). Coherent
Exciton Delocalization in Strongly Coupled Quantum Dot Arrays. Nano Lett..

[19] Tao A., Sinsermsuksakul P., Yang P. (2007). Tunable Plasmonic Lattices of Silver
Nanocrystals. Nat. Nanotechnol.

[20] Mueller N. S., Okamura Y., Vieira B. G. M., Juergensen S., Lange H., Barros E. B., Schulz F., Reich S. (2020). Deep Strong
Light–Matter Coupling in Plasmonic Nanoparticle Crystals. Nature.

[21] Lee Y. H., Lay C. L., Shi W., Lee H. K., Yang Y., Li S., Ling X. Y. (2018). Creating
Two Self-Assembly Micro-Environments to Achieve
Supercrystals with Dual Structures Using Polyhedral Nanoparticles. Nat. Commun..

[22] Alvarez-Puebla R. A., Agarwal A., Manna P., Khanal B. P., Aldeanueva-Potel P., Carbó-Argibay E., Pazos-Pérez N., Vigderman L., Zubarev E. R., Kotov N. A., Liz-Marzán L. M. (2011). Gold Nanorods
3D-Supercrystals as Surface Enhanced Raman Scattering Spectroscopy
Substrates for the Rapid Detection of Scrambled Prions. Proc. Natl. Acad. Sci. U. S. A..

[23] Sekh T. V., Cherniukh I., Kobiyama E., Sheehan T. J., Manoli A., Zhu C., Athanasiou M., Sergides M., Ortikova O., Rossell M. D., Bertolotti F., Guagliardi A., Masciocchi N., Erni R., Othonos A., Itskos G., Tisdale W. A., Stöferle T., Rainò G., Bodnarchuk M. I., Kovalenko M. V. (2024). All-Perovskite Multicomponent Nanocrystal Superlattices. ACS Nano.

[24] Li X., Chen L., Mao D., Li J., Xie W., Dong H., Zhang L. (2024). Low-Threshold Cavity-Enhanced
Superfluorescence
in Polyhedral Quantum Dot Superparticles. Nanoscale
Adv..

[25] Tian X., Chang H., Dong H., Zhang C., Zhang L. (2023). Fluorescence
Resonance Energy Transfer Properties and Auger Recombination Suppression
in Supraparticles Self-Assembled from Colloidal Quantum Dots. Inorganics (Basel).

[26] Liu J., Guillemeney L., Abécassis B., Coolen L. (2020). Long Range Energy Transfer
in Self-Assembled Stacks of Semiconducting Nanoplatelets. Nano Lett..

[27] Luo D., Qin X., Song Q., Qiao X., Zhang Z., Xue Z., Liu C., Mo G., Wang T. (2017). Ordered Superparticles with an Enhanced
Photoelectric Effect by Sub-Nanometer Interparticle Distance. Adv. Funct Mater..

[28] Montanarella F., Biondi M., Hinterding S. O. M., Vanmaekelbergh D., Rabouw F. T. (2018). Reversible Charge-Carrier
Trapping Slows Förster
Energy Transfer in CdSe/CdS Quantum-Dot Solids. Nano Lett..

[29] Montanarella F., Altantzis T., Zanaga D., Rabouw F. T., Bals S., Baesjou P., Vanmaekelbergh D., van Blaaderen A. (2017). Composite
Supraparticles with Tunable Light Emission. ACS Nano.

[30] Lam C. C., Leung P. T., Young K. (1992). Explicit Asymptotic Formulas for
the Positions, Widths, and Strengths of Resonances in Mie Scattering. Journal of the Optical Society of America B.

[31] Cai L., Pan J., Zhao Y., Wang J., Xiao S. (2020). Whispering Gallery
Mode Optical Microresonators: Structures and Sensing Applications. physica status solidi (a).

[32] Vanmaekelbergh D., van Vugt L. K., Bakker H. E., Rabouw F. T., de Nijs B., van Dijk-Moes R. J. A., van Huis M. A., Baesjou P. J., van Blaaderen A. (2015). Shape-Dependent
Multiexciton Emission and Whispering Gallery Modes in Supraparticles
of CdSe/Multishell Quantum Dots. ACS Nano.

[33] Neuhaus S.
J., Marino E., Murray C. B., Kagan C. R. (2023). Frequency Stabilization
and Optically Tunable Lasing in Colloidal Quantum Dot Superparticles. Nano Lett..

[34] Marino E., Bharti H., Xu J., Kagan C. R., Murray C. B. (2022). Nanocrystal
Superparticles with Whispering-Gallery Modes Tunable through Chemical
and Optical Triggers. Nano Lett..

[35] le
Feber B., Prins F., De Leo E., Rabouw F. T., Norris D. J. (2018). Colloidal-Quantum-Dot Ring Lasers with Active Color
Control. Nano Lett..

[36] Zhang C., Dong H., Zhang C., Fan Y., Yao J., Zhao Y. S. (2021). Photonic Skins Based on Flexible
Organic Microlaser
Arrays. Sci. Adv..

[37] Reale M., Marino E., Maçôas E., Ciccarello F., Cannas M., Cruz C. M., Campaña A. G., Sciortino A., Messina F. (2024). Carbon-Based Photonic Microlabels
Based on Fluorescent Nanographene-Polystyrene Composites. Adv. Funct Mater..

[38] Klimov V. I. (2000). Optical
Nonlinearities and Ultrafast Carrier Dynamics in Semiconductor Nanocrystals. J. Phys. Chem. B.

[39] Li Z., Wei J., Wang F., Tang Y., Li A., Guo Y., Huang P., Brovelli S., Shen H., Li H. (2021). Carrier Dynamics
in Alloyed Chalcogenide Quantum Dots and Their Light-Emitting Devices. Adv. Energy Mater..

[40] Yoshiki W., Tanabe T. (2014). All-Optical Switching Using Kerr Effect in a Silica
Toroid Microcavity. Opt Express.

[41] Pelc J. S., Rivoire K., Vo S., Santori C., Fattal D. A., Beausoleil R. G. (2014). Picosecond
All-Optical Switching in Hydrogenated Amorphous
Silicon Microring Resonators. Opt Express.

[42] Zhu T., Snaider J. M., Yuan L., Huang L. (2019). Ultrafast Dynamic Microscopy
of Carrier and Exciton Transport. Annu. Rev.
Phys. Chem..

[43] Zhu Y., Cheng J.-X. (2020). Transient Absorption
Microscopy: Technological Innovations
and Applications in Materials Science and Life Science. J. Chem. Phys..

[44] Maiuri M., Garavelli M., Cerullo G. (2020). Ultrafast Spectroscopy: State of
the Art and Open Challenges. J. Am. Chem. Soc..

[45] Schnedermann C., Lim J. M., Wende T., Duarte A. S., Ni L., Gu Q., Sadhanala A., Rao A., Kukura P. (2016). Sub-10 Fs Time-Resolved
Vibronic Optical Microscopy. J. Phys. Chem.
Lett..

[46] Guo Z., Manser J. S., Wan Y., Kamat P. V., Huang L. (2015). Spatial and
Temporal Imaging of Long-Range Charge Transport in Perovskite Thin
Films by Ultrafast Microscopy. Nat. Commun..

[47] Li S., Hu F., Bi Y., Yang H., Lv B., Zhang C., Zhang J., Xiao M., Wang X. (2023). Micrometer-Scale Carrier
Transport in the Solid Film of Giant CdSe/CdS Nanocrystals Imaged
by Transient Absorption Microscopy. Nano Lett..

[48] Huang L., Wong C., Grumstrup E. (2020). Time-Resolved Microscopy: A New Frontier
in Physical Chemistry. J. Phys. Chem. A.

[49] Yuan L., Chung T.-F., Kuc A., Wan Y., Xu Y., Chen Y. P., Heine T., Huang L. (2018). Photocarrier Generation
from Interlayer Charge-Transfer Transitions in WS _2_ -Graphene
Heterostructures. Sci. Adv..

[50] Mann S. A., Sciacca B., Zhang Y., Wang J., Kontoleta E., Liu H., Garnett E. C. (2017). Integrating
Sphere Microscopy for Direct Absorption
Measurements of Single Nanostructures. ACS Nano.

[51] Sauvan C., Hugonin J. P., Maksymov I. S., Lalanne P. (2013). Theory of the Spontaneous
Optical Emission of Nanosize Photonic and Plasmon Resonators. Phys. Rev. Lett..

[52] Montanarella F., Urbonas D., Chadwick L., Moerman P. G., Baesjou P. J., Mahrt R. F., van Blaaderen A., Stöferle T., Vanmaekelbergh D. (2018). Lasing Supraparticles Self-Assembled
from Nanocrystals. ACS Nano.

[53] Klimov V. I. (2007). Spectral
and Dynamical Properties of Multiexcitons in Semiconductor Nanocrystals. Annu. Rev. Phys. Chem..

[54] Chiasera A., Dumeige Y., Féron P., Ferrari M., Jestin Y., Nunzi Conti G., Pelli S., Soria S., Righini G. C. (2010). Spherical
Whispering-gallery-mode Microresonators. Laser
Photon Rev..

[55] Gérard J. M., Barrier D., Marzin J. Y., Kuszelewicz R., Manin L., Costard E., Thierry-Mieg V., Rivera T. (1996). Quantum Boxes as Active Probes for Photonic Microstructures:
The Pillar Microcavity Case. Appl. Phys. Lett..

