# Supplementary material for: Ultrafast Switching of Whispering Gallery Modes in Quantum Dot Superparticles
Source: Nano Lett. 2025 Feb 24;25(14):5828–35. doi: 10.1021/acs.nanolett.5c00643 (PMC12180688; doi:10.1021/acs.nanolett.5c00643)
Supplement: Supplementary file 1 [file nl5c00643_si_001.pdf]

SUPPLEMENTARY INFORMATION

For

Ultrafast Switching of Whispering Gallery Modes in Quantum Dot Superparticles

Pietro Castronovo[1], Marco Reale[1], Susan A. Rigter[2,3], Cherie R. Kagan[4,5,6], Christopher B. Murray[4,5], Salvatore Lorenzo[1], Erik C. Garnett[2,3], Peter Schall[3], Emanuele Marino[1], Alice Sciortino[1,7]*, Fabrizio Messina[1,7]*

[1] Dipartimento di Fisica e Chimica−Emilio Segrè, Università degli Studi di Palermo, Via Archirafi 36, 90123 Palermo, Italy

[2] Center for Nanophotonics, AMOLF, Science Park 104, 1098XG Amsterdam, The Netherlands

[3] Van der Waals−Zeeman Institute, University of Amsterdam, Science Park 904, 1098XH Amsterdam, The Netherlands

[4] Department of Chemistry, University of Pennsylvania, 231 S. 34th St., Philadelphia, Pennsylvania 19104, United States

[5] Department of Materials Science and Engineering, University of Pennsylvania, 220 S 33rd St., Philadelphia, Pennsylvania 19104, United States

[6] Department of Electrical and Systems Engineering, University of Pennsylvania, 200 S. 33rd Street, Philadelphia, Pennsylvania 19104 United States

[7] ATeN Center – Università degli Studi di Palermo, Viale delle Scienze, Edificio 18, 90128, Palermo, Italy

alice.sciortino02@unipa.it
fabrizio.messina@unipa.it

1 Materials and Methods

1.1 QD synthesis

CdSe nanocrystals with a first exciton peak at 568 nm were synthesized at 372 C for 40 s according to the literature[1]. A shell of 8 monolayers of CdS was grown on these CdSe nanocrystals by following the literature[2]. These protocols were followed with minor modifications outlined in detail in our recent paper[3]. The CdSe/CdS nanocrystals were dispersed in toluene at a concentration of 2 mg/mL for the assembly.

1.2 Assembly and structural characterization of QD Superparticles

Spherical superparticles of CdSe/CdS nanocrystals were synthesized by following the literature[3]. Briefly, a glass microfluidic chip is used to generate a oil in water emulsion. The dispersed phase of the emulsion consists of a dispersion of CdSe/CdS nanocrystals in toluene at 2 mg/mL, and the continuous phase of the emulsion consists of a 20 mM aqueous solution of sodium dodecyl sulphate. After generating the droplets, this "source" emulsion is mixed with a secondary "sink" emulsion consisting of 1% hexadecane in a 20 mM aqueous solution of sodium dodecyl sulphate. The sink emulsion is prepared separately by extended tip sonication, see paper for additional details[3]. Once mixed, the two emulsions flow together for 5 minutes at 70 C before being collected in a scintillation vial filled with 5 mL of a 20 mM aqueous solution of sodium dodecyl sulphate. During this time, the sink emulsion removes toluene from the source emulsion, causing the formation of superparticles. The vial is left uncapped on a hot plate set at 50 C for overnight, then the superparticles are washed thrice by mild centrifugation (100 g, 5 minutes) and redispersion in 10 mM aqueous solution of sodium dodecyl sulphate.

The superparticles were prepared for optical measurements by drop casting 10 uL of the dispersion on a substrate, followed by vacuum drying. The excess surfactant was removed by dipping the substrate in a mixture of isopropanol and water, 2:1 by volume, followed by vacuum drying.

1.3 UV-Vis ensemble measurements

Ensemble UV-Vis measurements on the diluted colloidal dispersion of QDs were performed at room temperature via an AVANTES optical fiber spectrophotometer based on a multi-channel CMOS detector (resolution 1.0nm), employing a dual source deuterium/halogen lamp.

1.4 Micro-UV-Vis measurements

Micro-UV-Vis (µ-UV-VIS) measurements on single superparticles were performed on the integrating sphere microscopy setup described in[4]. The sample, deposited on a 1 mm thick glass substrate, is placed inside a custom built integrating sphere, and a monochromatized supercontinuum laser is tightly focused (approx. 1µm) over a single superparticle via a long working distance objective. As thoroughly discussed in [4], reflected and scattered light are then measured as a function of the wavelength, with a resolution of 3nm. The recorded intensities are subsequently combined to obtain the absorptance A of the sample, defined as the ratio of absorbed to incident power, that is:

$$A(\lambda) = \frac{P_{abs}}{P_{in}}$$

In order to allow for a direct comparison with steady-state OA and pump/probe data, to the purposes of this work the absorptance data were converted into optical density through the following relation

$$OD(\lambda) = \log_{10}\frac{P_{in}}{P_t} = -\log_{10}(1 - A(\lambda))$$

1.5 Ensemble Photoluminescence measurements

Ensemble PL spectra were obtained via a VARIAN CARY Eclipse spectrofluorometer, employing a Xenon lamp as the excitation source and collecting the PL emission in a right angle geometry. The excitation wavelength has been fixed at 400nm for all measurements, whereas the collection scanning speed, integration time and number of averages have been optimized in order to maximize the signal-to-noise ratio, obtaining a resolution of 1.5nm.

1.6 Micro-Photoluminescence measurements

Micro-photoluminescence (μPL) measurements on single SPs were performed by means of a modular setup assembled from an excitation laser source (405 nm CW laser diode, Thorlabs), an optical microscope (Motic BA300) and a spectrophotometer (Acton SpectraPro 2300i monochromator coupled to a PI Pixis 400 CCD camera). The sample, drop-casted on a 1mm thick glass slide, is photoexcited by the focused beam of a CW laser diode, in a reflection wide-field geometry. An optical fiber (1 mm core diameter) positioned on the image plane of the 40X/0.65 microscope objective, is used to collect the emission from a specific region of the sample, selected in such a way to contain only one superparticle (bright yellow circle in Figure S2a). The collected light is subsequently dispersed by the monochromator and recorded by the CCD camera. Fluorescence images were taken by coupling a camera to one of the oculars of the microscope while filtering out the scattered excitation light via a long-pass filter. Scale calibration was performed by acquiring an image of a test pattern (Thorlabs) in the same experimental condition as the measurements.

1.7 Micro-Pump-Probe measurements

Ultrafast Micro-Pump-Probe (μPP) measurements were performed via the home-built setup schematized in Figure S3a, pumped via the 50 fs, 800nm laser pulses (FWHM=30 nm, 350 mJ energy per pulse) produced by a 5 kHz Ti:sapphire femtosecond amplifier (Spectra Physics Solstice-Ace). Said pulses are split 80%/20% via a beam splitter in order to generate the 400 nm pump and broadband probe respectively. The former is obtained via second harmonic generation (20% efficiency, 50-100 nJ/pulse, later attenuated in order to achieve the desired power) in a 250 μm β-BBO crystal and subsequently isolated from the residual fundamental by a Schott BG40 filter and chopped at 500 Hz, whereas the latter is obtained by focusing the 800 nm beam on a 1 mm quartz cell containing D_2O, generating a "white light" pulse extending from 400 nm to 750 nm. Both pump and probe are subsequently tightly focused on the sample (deposited on a 0.07mm thick glass substrate) via a parabolic mirror with 20 mm effective focal length. In this configuration, the probe beam can be focused down to 2μm FWHM (as discussed below), whereas the size of the pump beam can be set in the range 2-15 μm FWHM by changing the beam cross section before the parabolic mirror. After interacting with the sample, the probe is collected by a 40X/0.65 microscope objective coupled with a tube lens (Thorlabs TTL180-A, focal length 180mm), spectrally dispersed by a grating-based monochromator (spectral resolution of 0.5nm) and focused on a 1024-pixel detector (Glaz Linescan-I) with single shot capabilities. Optical micrographs of the sample can be obtained by illuminating it with white light from a lamp, reflected by the same parabolic mirror used for μPP measurements, and collecting the transmitted light on the focal plane of the tube lens via a monochrome CMOS camera. Before performing the experiment, the focal planes of the parabolic mirror and microscope objective are matched by minimizing the size of the probe spot as seen by the above-mentioned camera, which is subsequently used in order to perform the overlap procedure described in Figure S3. In a typical measurement, the signal is obtained by averaging 5000 pumped and unpumped spectra for each pump-probe delay and scanning over the latter a minimum of 10 times. The raw data is then subjected to correction procedures aimed at eliminating the effects of cross-phase modulation (XPM) and group velocity dispersion (GVD)[5], thereby obtaining the final data presented in the paper. The spatial and temporal resolutions of the setup were determined as follows.

Spatial resolution: the spatial resolution of the setup is determined by the size of the probe spot at the focal plane of the parabolic mirror (corresponding to the sample plane). This can be readily obtained by measuring the probe spot at the focal plane of the tube lens via a beam profiling camera as shown in Figure S3b. From the resulting image, horizontal and vertical cuts were then performed as indicated by the magenta and orange cross-hairs, obtaining the traces in panels c-d of Figure S3. Each trace was subsequently fitted with a gaussian curve, whose full width at half maximum (FWHM) yields an estimate of the size D of the spot in each direction, obtaining $D_{hor} = (93 \pm 6)\mu m$ and $D_{vert} = (97 \pm 6)\mu m$ and therefore an average size $D = (95 \pm 6)\ \mu m$. Keeping in mind the 40X magnification of the objective, the probe spot size at the sample plane can finally be obtained as $d = \frac{D}{40} = (2.37 \pm 0.15)\ \mu m$.

Temporal resolution: The temporal resolution, or instrumental response function (IRF), of the setup is determined by the duration of the pump pulse. Other effects which can degrade temporal resolution, such as group velocity mismatch within the sample or non-collinear geometrical effects, can be totally neglected here as the sample is very thin. For pulses characterized by a gaussian temporal envelope, the IRF can be effectively obtained from the XPM signal by assuming the full duration Δt of the latter to be approximately equal to 6σ, where σ is the standard deviation of the gaussian envelope. The IRF can therefore be estimated as the FWHM of the envelope, that is $2.35482\sigma = \frac{2.35482}{6}\Delta t$.

Figure S3e reports the μPP signal of a single SP excited with 400nm pump pulses at a fluence of $2.8\ mJ/cm^2$. Such fluence, higher than that employed for the measurements reported in the main paper,

allows for a clear visualization of the XPM signal, the total duration of which can be estimated as $\Delta t \simeq 130\,fs$ (within the two green lines). This corresponds to a standard deviation $\sigma \simeq 21.7\,fs$ and a temporal resolution (FWHM) of $2.35482\sigma \simeq 51\,fs$. Notably, this is almost identical to the nominal pulse duration guaranteed by the source.

1.8 Absolute Quantum Yield (QY) measurements

The absolute photoluminescence quantum yields (QYs) of QDs and SPs were determined in an integrating sphere following standards protocols in the literature[6].
More in detail, the colloidal dispersions of QDs and SBs were placed in quartz NMR tubes, positioned inside a LabSphere integrating sphere and illuminated in indirect excitation geometry via a Thorlabs CW laser diode at 405nm. The integrating sphere was coupled to an optical fiber spectrometer, enabling the acquisition of the spectral distribution of the emission (P) and unabsorbed laser light (L) intensities. Identical measurements were then performed on pure solvent (toluene for QDs, SDS in water for SBs), in order to obtain the full incident laser intensity (L_0). The QY was finally obtained as $\eta = 100 \times \frac{P}{L_0 - L}$.
It is important to note that, while the QDs form a highly stable colloidal suspension, the much larger SPs tend to precipitate quite quickly. Therefore, the colloidal suspension of SBs was subjected to a minimum of 30 s vortex mixing, and the QY measurements were performed swiftly thereafter in order to avoid precipitation.

1.9 Theoretical simulations – light scattering by a gaussian beam

In order to understand how the probe beam interacts with a QD superparticle within our µPP setup, we performed theoretical simulations of the scattering of a gaussian beam (2µm FWHM) with a dielectric sphere of radius a=5.4µm, whose refractive index was obtained as the weighted average of the refractive indexes of its components, namely CdSe, CdS and oleate ligands.

Following[7] we use the Bromwich formalism[8] to solve the Maxwell equations, taking into account boundary conditions, in spherical coordinate systems. The solution is written as the sum of two special solutions: the transversal Magnetic wave (TM) and the transverse Electric wave (TE). The TE ($H_r = 0$) and TM ($E_r = 0$) -fields are obtained from Bromwich Scalar Potentials (BSP) UTE and UTM through the following equation

$$\frac{\partial^2 U}{\partial r^2} + k^2 U + \frac{1}{r^2 \sin\theta}\frac{\partial}{\partial\theta}\left(\sin\theta\frac{\partial U}{\partial\theta}\right) + \frac{1}{r^2 \sin\theta^2}\frac{\partial^2 U}{\partial\phi^2} = 0$$

where k is the wave number.

Cartesian description - Assume a potential vector polarized in the transverse direction $A = \left(\psi e^{-ikz}, 0{,}0\right)$ and let w_0 be the width parameter at the waist and $l = kw_0^2$ the diffraction length. We introduce the dimensionless variables $(\tilde{x}, \tilde{y}, \tilde{z})$: $x = w_0\tilde{x}$, $y = w_0\tilde{y}$, $z = l\tilde{z}$ and a fundamental dimensionless parameter

$$s = \frac{w_0}{l} = \frac{1}{kw_0}$$

The function ψ is expanded as

$$\psi = \psi_0 + s^2\psi_2 + s^4\psi_4 + \cdots$$

The electric components are then found to be

$$E_x = E_0\left[\psi_0 + s^2\left(\psi_2 + \frac{\partial^2\psi_0}{\partial\tilde{x}^2}\right) + \cdots\right]e^{-ikz}$$

$$E_y = E_0\left[s^2\frac{\partial^2\psi_0}{\partial\tilde{x}\,\partial\tilde{y}} + s^4\frac{\partial^2\psi_2}{\partial\tilde{x}\,\partial\tilde{y}} + \cdots\right]e^{-ikz}$$

$$E_z = E_0\left[-is\frac{\psi_0}{\partial\tilde{x}} - is^3\left(\frac{\partial\psi_2}{\partial\tilde{x}} + i\frac{\partial^2\psi_0}{\partial\tilde{x}\,\partial\tilde{z}}\right) + \cdots\right]e^{-ikz}$$

Where the lowest order ψ_0 is the fundamental mode solution $\psi_0 = e^{-iP+Q(\tilde{x}^2+\tilde{y}^2)}$ with $iP = -\ln i\,Q$ and $Q = 1/(i + 2\tilde{z})$.

The BSP for external wave - The BSP for the external waves are given by[7]:

$$U_{TM}^{e} = \frac{E_0}{k^2}\cos\phi\sum_{n=1}^{\infty} i^{n+1}(-1)^n\frac{2n+1}{n(n+1)}g_n a_n \xi_n(kr)P_n^1(\cos\theta)$$

$$U_{TE}^{e} = \frac{H_0}{k^2}\sin\phi\sum_{n=1}^{\infty} i^{n+1}(-1)^n\frac{2n+1}{n(n+1)}g_n b_n \xi_n(kr)P_n^1(\cos\theta)$$

where $P_n^1(\cos\theta)$ the associated Legendre polynomials and
the functions $\xi_n(kr)$ are chosen in order to satisfy the boundary conditions in the limit $r \to \infty$. They are defined by :

$$\xi_n(kr) = \psi_n(kr) + \mathrm{i}(-1)^n\left(\frac{\pi kr}{2}\right)^{1/2} J_{-n-1/2}(kr)$$

With the $J_{-n-1/2}$ are the Bessel functions of half-integer order.
The scattering coefficients are obtained by

$$a_n = \frac{\psi_n(\alpha)\psi_n'(\beta) - m\psi_n'(\alpha)\psi_n(\beta)}{\xi_n(\alpha)\psi_n'(\beta) - m\xi_n'(\alpha)\psi_n(\beta)}$$

$$b_n = \frac{m\psi_n(\alpha)\psi_n'(\beta) - \psi_n'(\alpha)\psi_n(\beta)}{m\xi_n(\alpha)\psi_n'(\beta) - \xi_n'(\alpha)\psi_n(\beta)}$$

where m is the complex refractive index of the particle, $\alpha = 2\pi r/\lambda$ and $\beta = m\alpha$.

The scattered field components – Given a_n and b_n, the scattered fields read:

$$E_r = E_0\cos\phi\sum_{n=1}^{\infty} i^{n+1}(-1)^n\frac{2n+1}{n(n+1)}g_n a_n[\xi_n''(kr) + \xi_n(kr)]P_n^1(\cos\theta)$$

$$E_\theta = \frac{E_0}{kr}\cos\phi\sum_{n=1}^{\infty} i^{n+1}(-1)^n\frac{2n+1}{n(n+1)}[g_n a_n\xi_n'(kr)\tau_n(\cos\theta) - ig_n b_n\xi_n(kr)\pi_n(\cos\theta)]$$

$$E_\phi = \frac{-E_0}{kr}\sin\phi \sum_{n=1}^{\infty} i^{n+1}(-1)^n \frac{2n+1}{n(n+1)}[g_n a_n \xi'_n(kr)\pi_n(\cos\theta) - i g_n b_n \xi_n(kr)\tau_n(\cos\theta)]$$

where the Legendre functions $\pi_n(\cos\theta)$and $\tau_n(\cos\theta)$ are defined by

$$\pi_n(\cos\theta) = \frac{P_n^1(\cos\theta)}{\sin\theta}$$

$$\tau_n(\cos\theta) = \frac{\mathrm{d}}{\mathrm{d}\theta}P_n^1(\cos\theta)$$

Scattered field intensities in the far-field – In the far-field ($r \gg \lambda$) the functions $\xi(kr)$ simplify[9] to $\xi(kr) = i^{n+1}e^{-ikr}$ and the scattered wave become:

$$E_\theta = \frac{iE_0\exp(-ikr)}{kr}\cos\phi \sum_{n=1}^{\infty}\frac{2n+1}{n(n+1)}g_n[a_n\tau_n(\cos\theta) + b_n\pi_n(\cos\theta)]$$

$$E_\phi = \frac{-\mathrm{i}E_0\exp(-ikr)}{kr}\sin\phi \sum_{n=1}^{\infty}\frac{2n+1}{n(n+1)}g_n[a_n\pi_n(\cos\theta) + b_n\tau_n(\cos\theta)]$$

Figure S4 reports the intensities of front- and back-scattered light at infinite distance as a function of angle. A simple comparison reveals that almost all the light is front-scattered at angles well within the acceptance cone of our microscope objective (NA=0.65). A rather straightforward calculation shows that, by collecting the totality of scattered light, any μPP signal must be the consequence of a genuine photoexcitation-dependent absorbance variation.

Indeed, let I_0 be the incident probe intensity, and $I_A, I_T, I_S(\Omega)$ the absorbed, transmitted, and scattered intensities, respectively. It immediately follows that:

$$I_0 = I_A + I_T + \int I_S(\Omega)d\Omega$$

where the wavelength dependence has been omitted for ease of notation.

Assuming that the entirety of scattered light is collected by the objective, we have:

$$I_{collected} = I_T + \int I_S(\Omega)d\Omega = I_0 - I_A$$

As explained above, the μPP signal is obtained by comparing the probe intensity registered through the photoexcited and unexcited system. More in detail, the differential absorbance $\Delta A(\lambda)$ is obtained as:

$$\Delta A(\lambda) = \frac{1}{\ln 10}\ln\frac{\left(I_{collected}(\lambda)\right)_U}{\left(I_{collected}(\lambda)\right)_P} = \frac{1}{\ln 10}\ln\frac{(I_0(\lambda) - I_A(\lambda))_U}{(I_0(\lambda) - I_A(\lambda))_P}$$

where the indexes U and P indicate the unexcited and photoexcited systems, respectively.

Because $I_0(\lambda)$ does not depend on photoexcitation, any non-zero $\Delta A(\lambda)$ must be the consequence of a difference between $(I_A(\lambda))_U$ and $(I_A(\lambda))_P$, that is to say, any µPP signal must stem from a photoinduced variation in the absorption profiles of the system.

2 Supplementary discussions

As mentioned in the main paper, the modulations observed in µPP can be linked to a shift in the spectral positions of WGM resonances, due in turn to a combination of electronic and thermal effect on the size and refractive index of the SP. Here we briefly discuss some aspects linked to this attribution

2.1 Impact of the absorbance on the quality factor of active microresonators

As reported in[10], the quality factor of an active microresonator at a given wavelength is inversely proportional to its absorption coefficient. In our case, using the tabulated absorption coefficient of CdS [SI_5], which is the main component of SPs by mass, we roughly estimate Q>10^3 at 550 nm, quickly going down to Q≃32 at 530 nm, because of the onset of CdS shell absorption, as clearly seen in Figure 1d.

2.2 WGM modulations and effective refractive index

To fully appreciate the spectral shape of the observed WGM modulations, it is crucial to keep in mind that the frequency structure of the WGMs is non-trivial and not expected to appear as a simple frequency comb with a single periodicity. As reported in the literature[10], one expects to observe modes with different values of the radial index i=1, 2, …. , each comprising two separate progressions (Transverse Electric and Transverse Magnetic modes) with slightly different periodicity from each other and depending on the radial index. Normally the visibility of WGMs rapidly goes down with increasing radial index because of increased losses, which tends to simplify the otherwise very complex structure of the resonator. To better highlight the frequency structure of our modulations, we performed a fast Fourier transform (FFT) of the modulation amplitude plotted as a function of energy (Figure S9a). From the FFT (Figure S9b), at least three peaks clearly emerge over the background: two of these peaks are very close to each other and are associated to the TE and TM progressions at i=1, while the third is most likely the frequency comb generated by modes with higher radial index (i=2). For the purposes of our study, we employed a simple, yet effective, approximation of the complex frequency structure described above, given by:

$$\nu_l \simeq \frac{cl}{2\pi a n}$$

where l is the number of internal reflections ($100 < l < 150$ in the spectral region covered by our probe), a is the radius of the microresonator and n is the real part of its average refractive index.

As shown in Figure S11, the positions of the negative modulation peaks extracted from the µPP signal form a very regular progression, confirming the validity of the approximation discussed above.

The slope of this progression represents the mode separation:

$$\Delta\nu \simeq \frac{c}{2\pi a n}$$

A simple linear fit yields:

$$\Delta\nu_{neg} = (4.03 \pm 0.03)THz$$

From Δν, using the known SP radius $a = (5.4 \pm 0.2)\ \mu m$ (here assumed constant), we can then estimate an effective, wavelength-averaged real part of the refractive index equal to:

$$n_{avg} = 2.19 \pm 0.02$$

2.3 Estimate of the overall radius/refractive index variation

We can obtain an estimate of the photoinduced change of $x = an$ by considering the average distance between a given negative peak and its closest positive counterpart. From the data shown in Figure S11a, we obtain:

$$d\nu_l \simeq -1.8\ THz$$

From theory, we expect:

$$d\nu_l \simeq -\frac{cl\Delta x}{2\pi x^2}$$

Comparing the two expressions, recalling the abovementioned values for the radius and effective refractive index of the SP and considering an intermediate value l=125 for the mode index, we can estimate the relative variation Δx/x as:

$$\frac{\Delta x}{x} = 0.0034 \pm 0.0013$$

which corresponds to a percentual variation of $(0.34 \pm 0.13)\%$.

2.4 Estimate of the photoinduced thermal change and the associated refractive index and radius variations

In order to obtain the pump-induced temperature variation of the SP, we first of all need to consider the penetration depth of the pump within the superparticle. This parameter can be estimated from the tabulated absorption coefficient of CdS (the main component of our SPs)[11], yielding $\delta a \simeq 100nm$.

Therefore, we can safely assume that the whole pump energy E_p is absorbed by the SP's outermost layer (of thickness δa), causing a temperature rise ΔT according to the law:

$$E_p = 4\pi\rho a^2 \delta a\ C\Delta T$$

where ρ and C indicate the density and specific heat of CdS[12]. By reversing this equation, we obtain:

$$\Delta T = \frac{E_p}{4\pi\rho a^2 \delta a\ C} \simeq 7K$$

Such a temperature rise will contribute to further increase Δn and hence the amplitude of the modulations. Indeed, by utilizing reported data for the temperature dependence of the refractive index of CdS[13] we estimate:

$$\frac{\Delta n}{\Delta T} \simeq 4 \times 10^{-4} K^{-1}$$

and therefore:

$$\Delta n = \frac{\Delta n}{\Delta T}\Delta T \simeq 0.003$$

This represents a sizeable portion of the overall variation we calculate above, confirming its partial thermal origin coherently with the partial (relatively) slow rise of the modulation amplitude we observe in Figure 3e and Figure S12.

On the other hand, the thermally-induced radius variation Δa_{th} can be estimated from the known (linear) thermal expansion coefficient of CdS[12]: $\alpha \simeq 4 \times 10^{-6} K^{-1}$. From this, considering a total temperature variation of $\simeq 7\ K$ and a volumetric expansion coefficient $\beta = 3\alpha \simeq 1.2\ \times 10^{-5} K^{-1}$, we obtain a diameter variation of our $\simeq 10\ \mu m$ SPs of about $0.3\ nm$. This variation is certainly negligible when compared to the refractive index variation.

3. Supplementary Figures

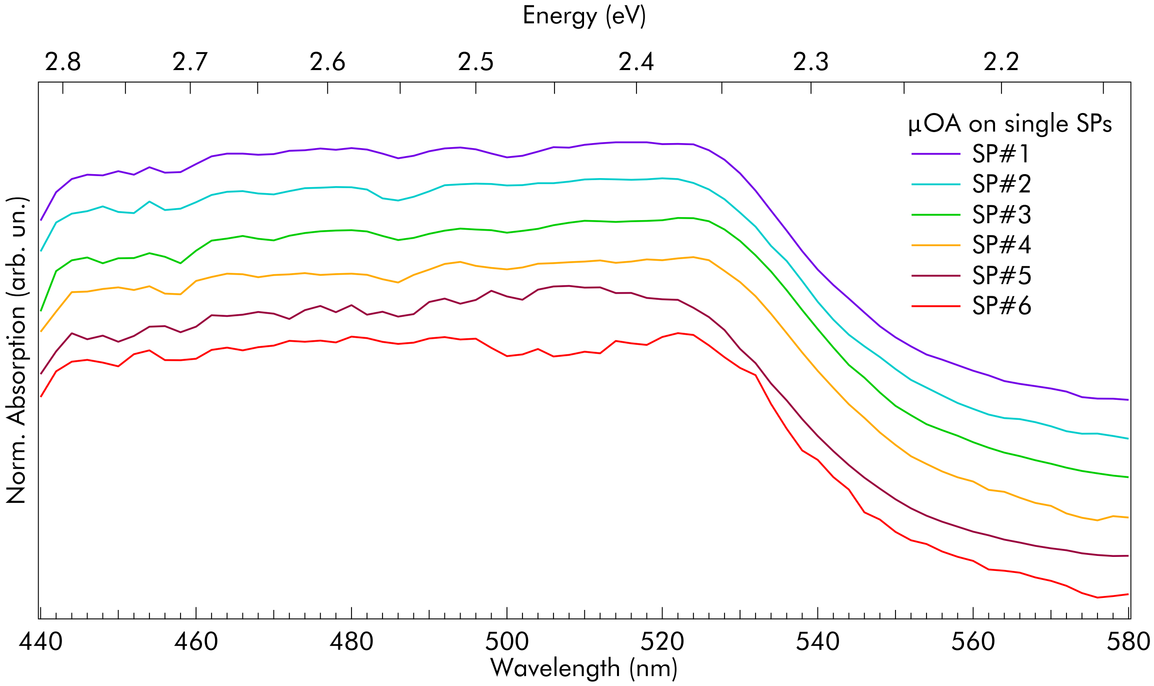

Figure S1: Normalised μ-UV-Vis spectra of six distinct QD Superparticles, obtained via integrating sphere microscopy. Each spectrum is vertically shifted with respect to the others to aid legibility.

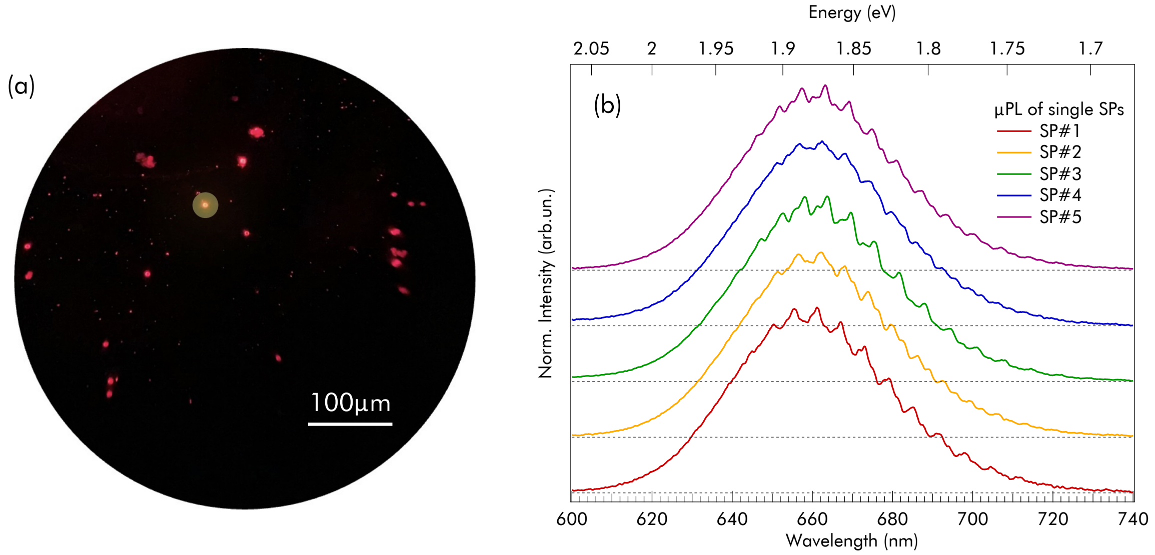

Figure S 2: (a) Fluorescence micrograph of QD Superparticles deposited on a glass slide. The yellow circle indicates the region from which PL emission is collected; (b) Normalised µPL spectra of five distinct SPs (vertically shifted for ease of reading), each displaying unique WGM resonance peaks.

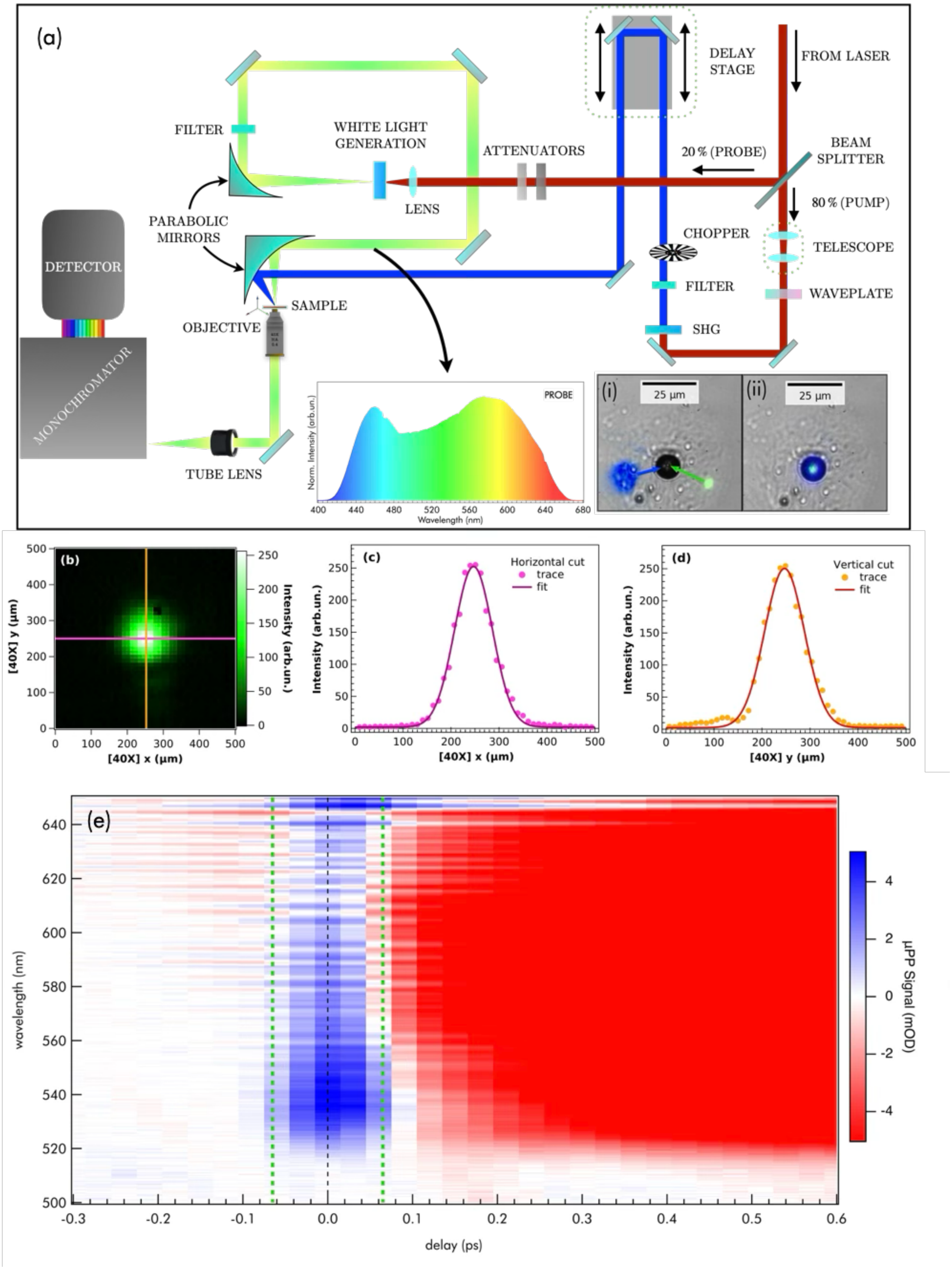

Figure S 3 : (a) schematic representation of the home-built micro Pump-Probe (µPP) setup. The insets show the typical spectrum of the white-light probe, ranging from 420 to 660nm, as well as a false-colour micrograph of the relative positions of the pump (blue) and probe (green) spots with respect to the superparticle before (i) and after (ii) the overlap procedure; (b-d) determination of the spatial resolution (probe spot size) of the setup: (a) false color micrograph of the probe as seen by a beam profiling camera placed on the image plane of the tube lens, (b-c) traces obtain by horizontal and vertical cuts of panel b, fitted with gaussian curves; (e) µPP signal at small delays of a single SP excited with 400nm pump pulses at 2.8mJ/cm^2 fluence, showcasing the XPM signal (within the two green dotted lines).

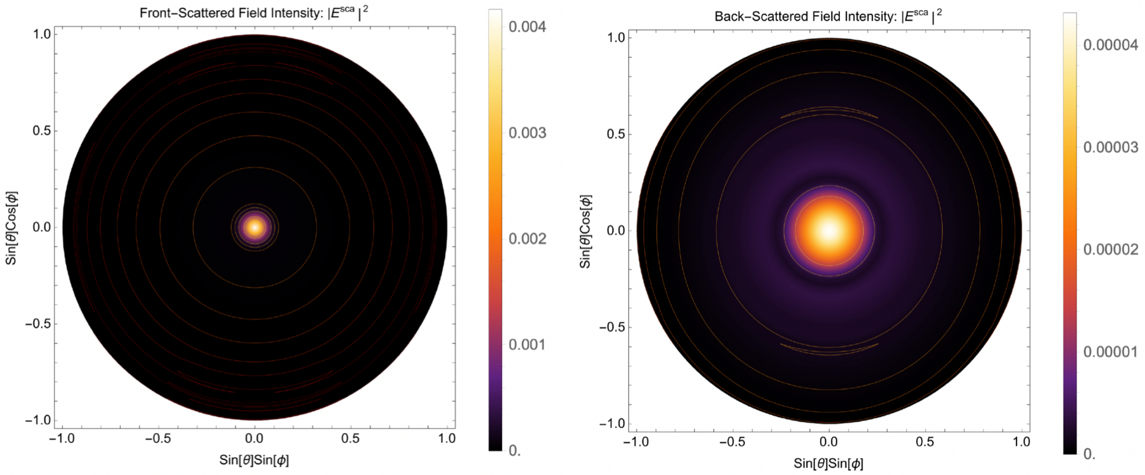

Figure S 4: Front-Scattered (left plot) and Back-Scattered (right plot) field intensity as obtained via the theoretical simulations described in the methods section.

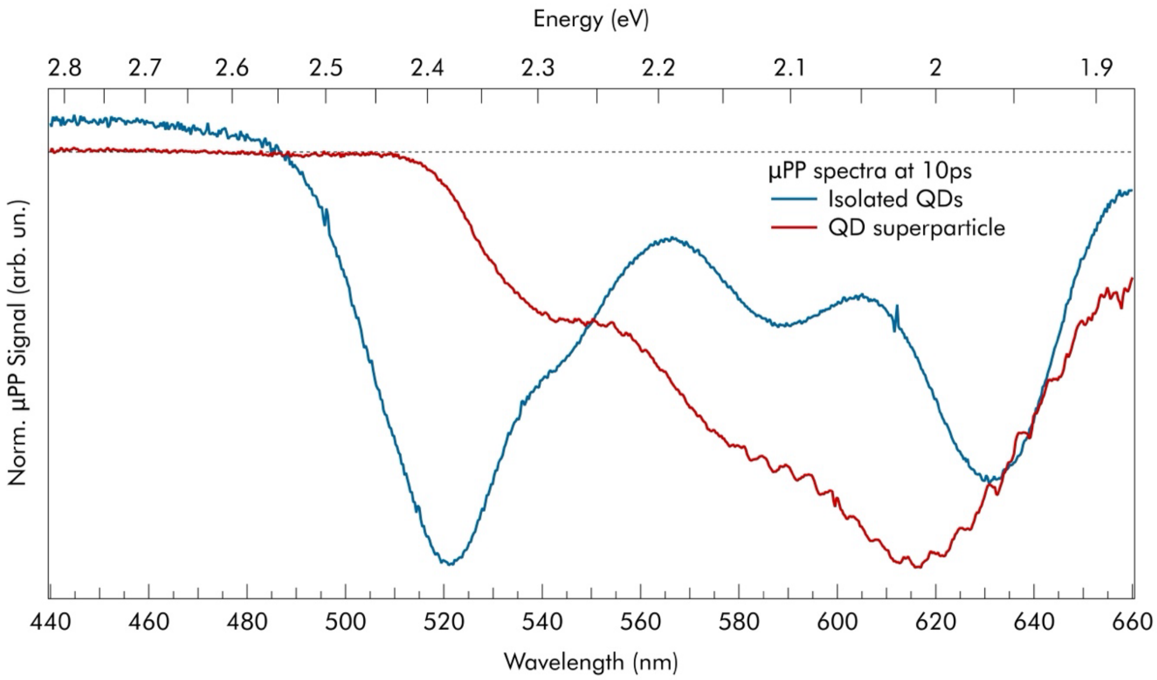

Figure S 5: Comparison between normalised µPP spectra at 10ps delay of a colloidal dispersion of CdSe/CdS QDs deposited on a substrate and a single QD Superparticle, highlighting the different spectral positions and relative intensities of the GSB signals as well as the absence of modulation in the former sample.

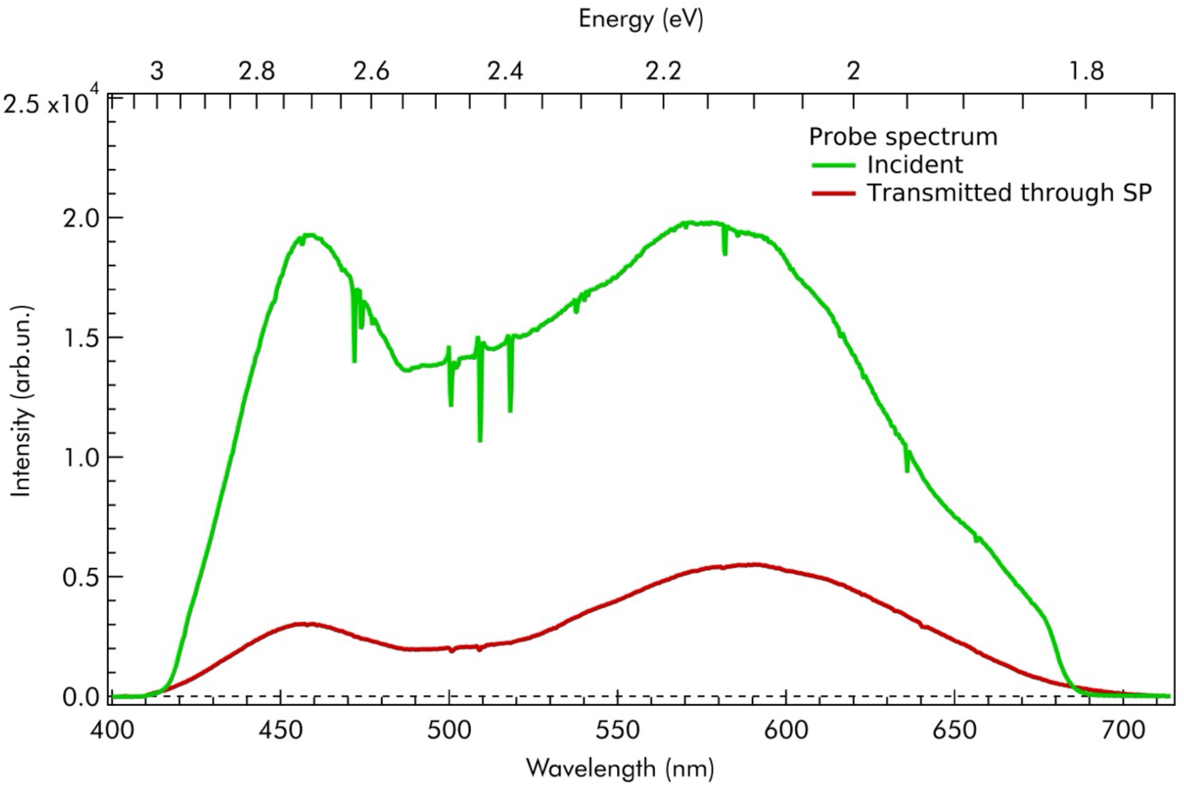

Figure S 6: Comparison between the spectral profiles of the incident probe light (green) and the probe light transmitted through a SP (red).

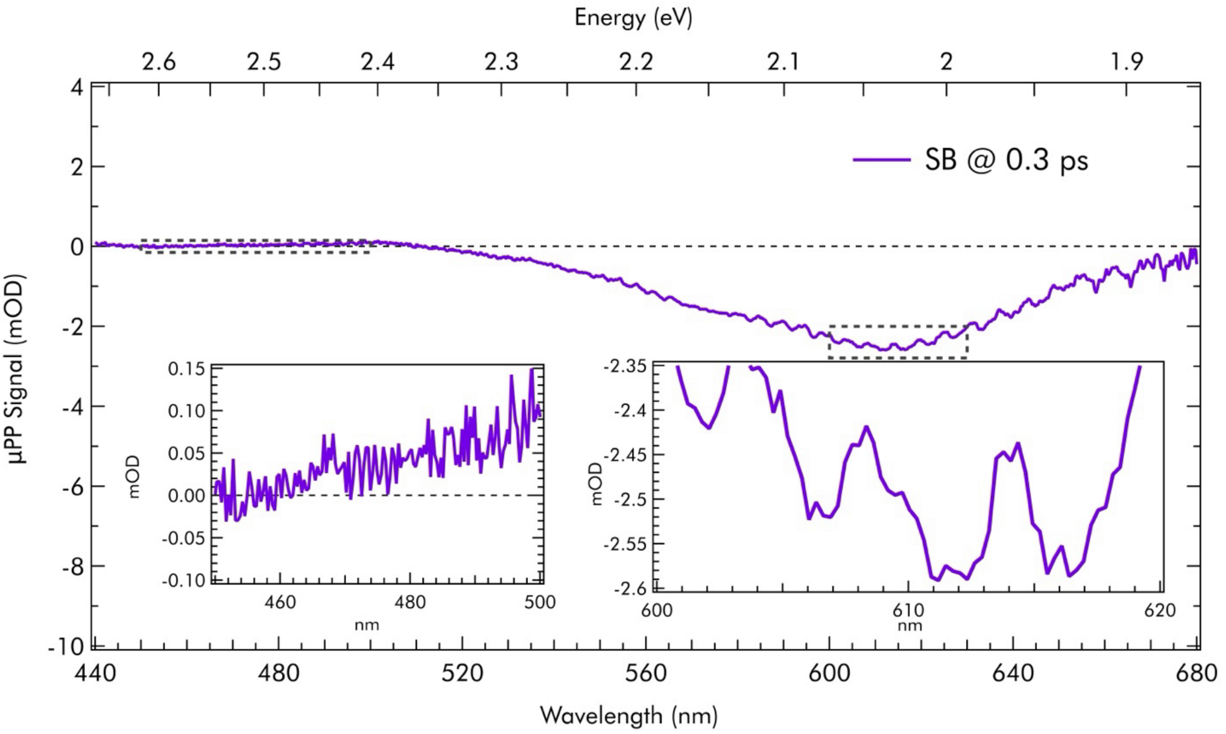

Figure S 7: µPP spectrum of a single SB at 0.3ps pump-probe delay. The insets show a comparison between the noise floor (left) and the modulations (right).

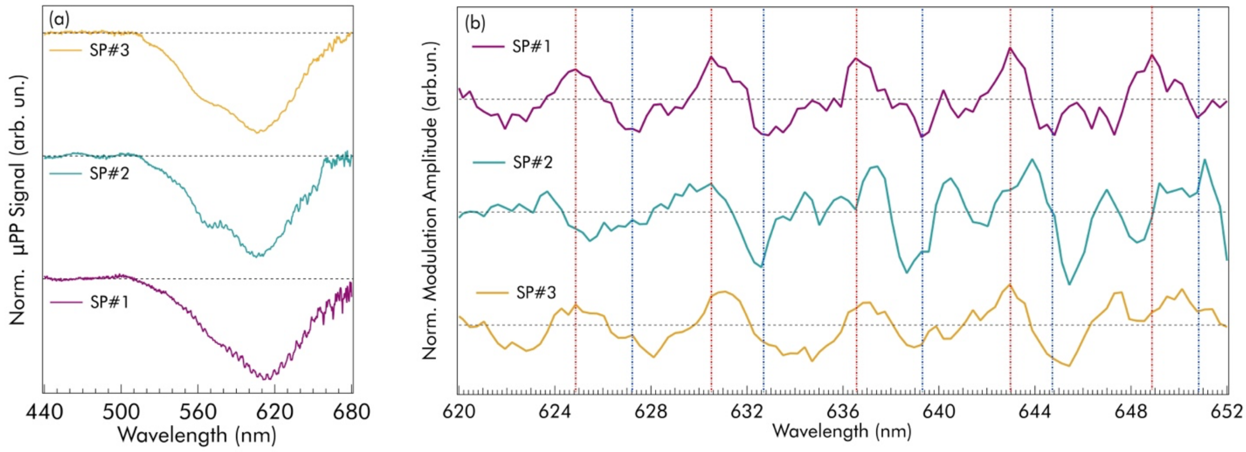

Figure S 8: (a) Normalized µPP spectra of SP#1, SP#2, and SP#3 at 0.3 ps pump-probe delay, vertically shifted in order to highlight the different relative intensities of the modulations (more visible in SP#1, less so in SP#2, almost imperceptible in SP#3); (b) Zoom of the extracted modulations (Figure 2f) in the range 620-652nm. The vertical lines pinpoint the positive (red) and negative (blue) lobes in the modulation of SP#1, showing the different positions of those of SP#2 and SP#3.

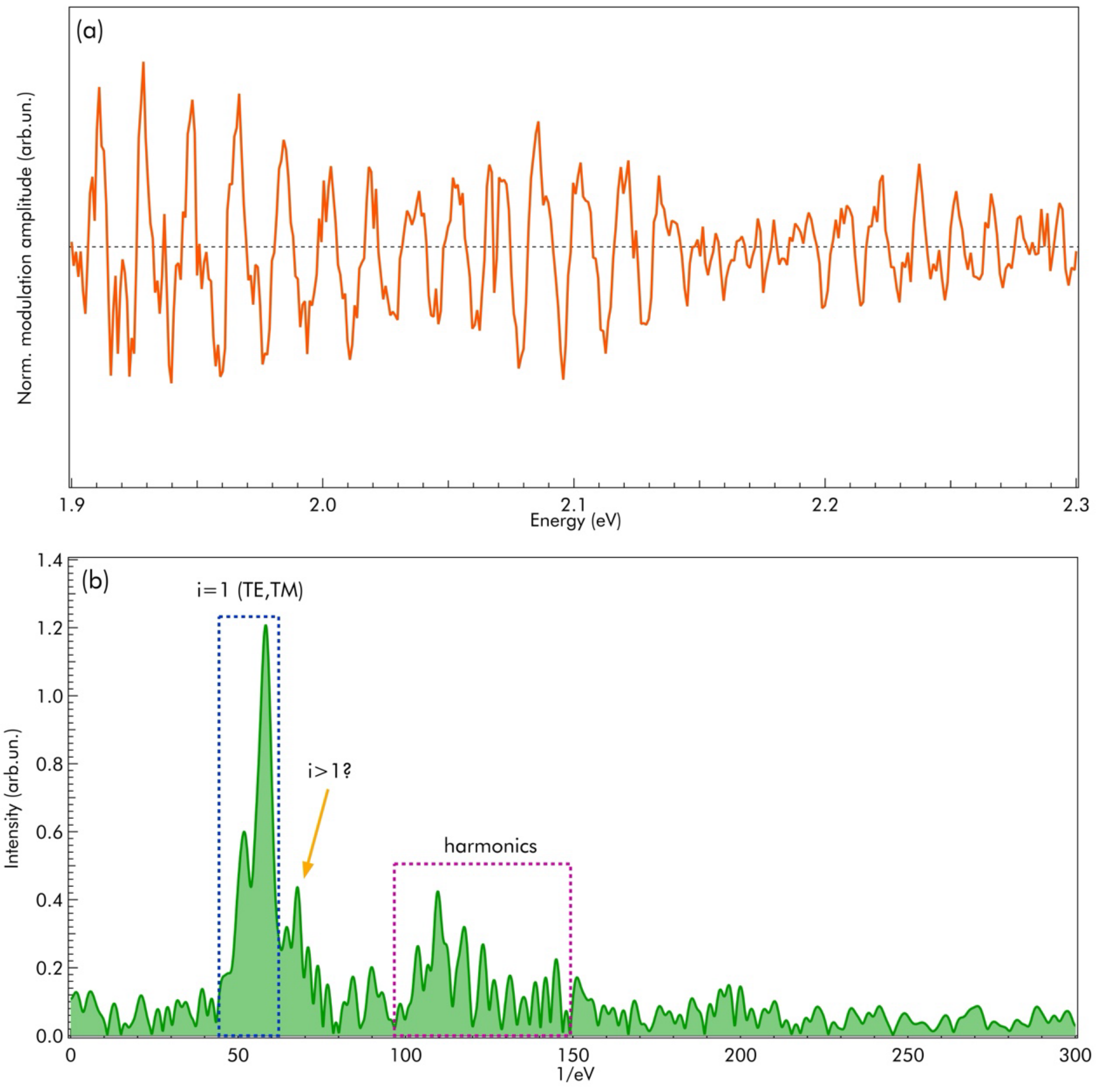

Figure S 9: (a) normalized modulation amplitude of SP#1 at 1 ps delay; (b) fast Fourier transform of the signal in (a). The boxes and arrow indicate the main visible peaks.

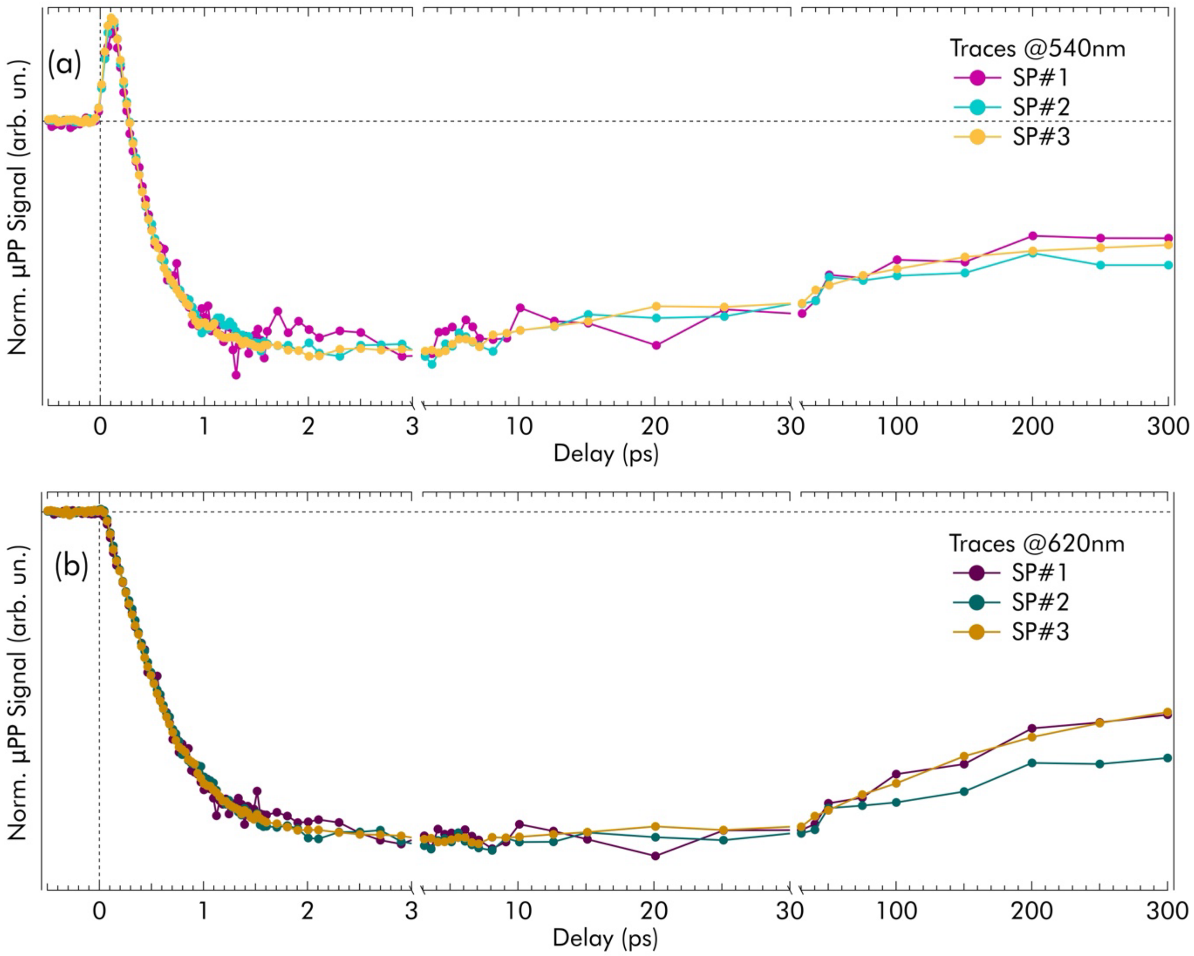

Figure S 10: Normalized µPP kinetic traces of three distinct SPs extracted at (a) 540nm (1P transition) and (b) 620nm (1S transition). In spite of the differences in spectral shape, the SPs display identical kinetics.

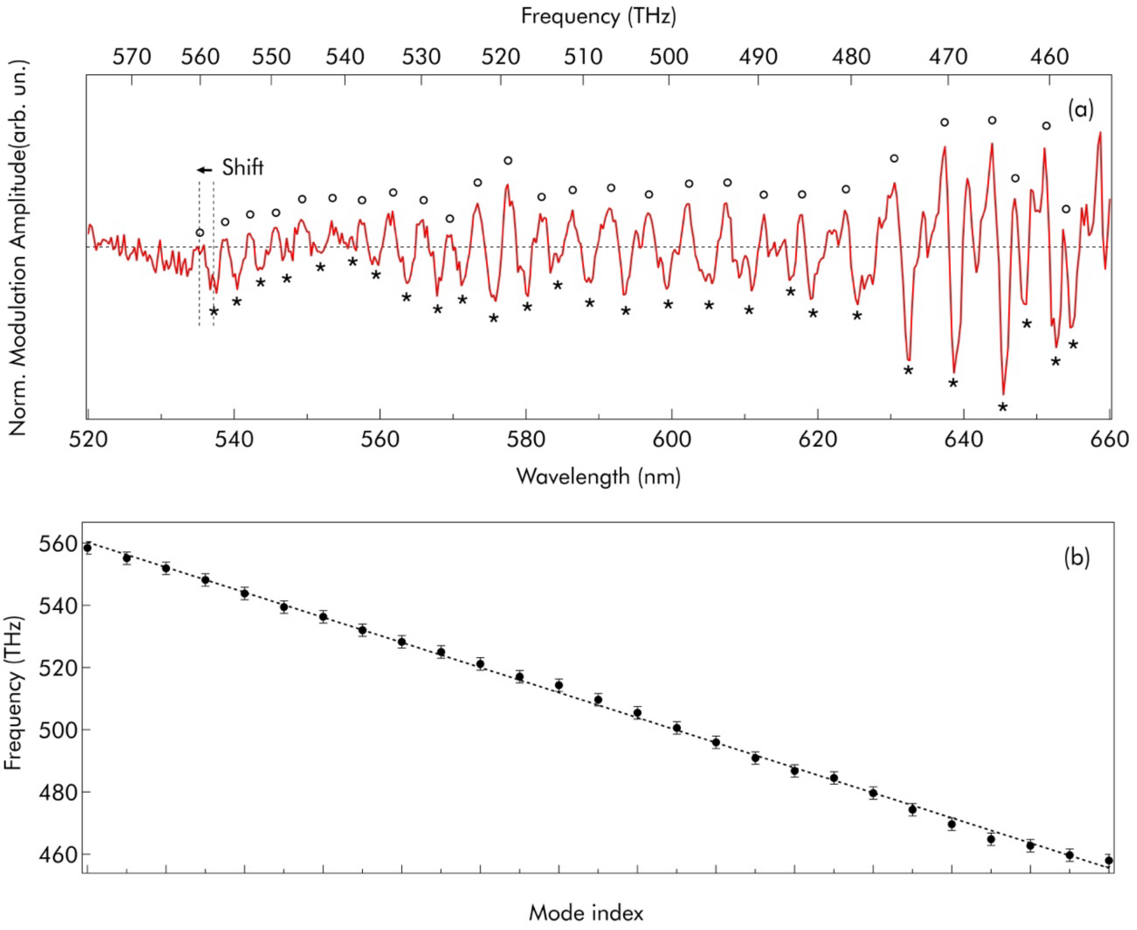

Figure S 11: (a) Modulations extracted from the µPP signal of a single SP as shown in Figure 3c; the circular and star-shaped markers highlight the positions of positive and negative peaks, respectively. (b) Frequency of each negative peak plotted as a function of the mode index. The dashed line indicates the linear fit on the progression.

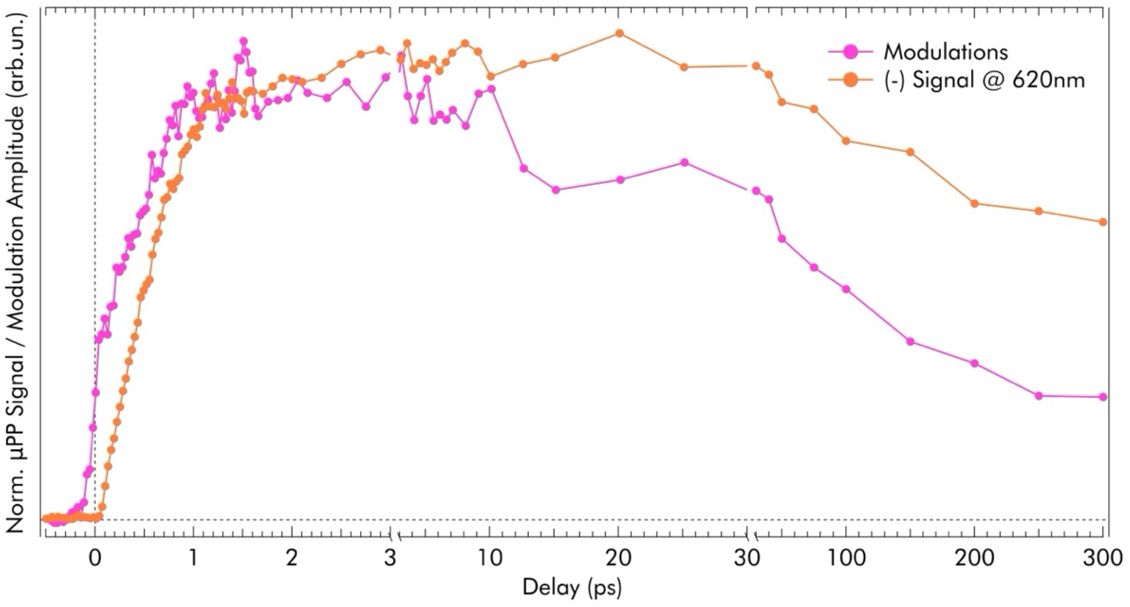

Figure S 12: comparison between the time evolution of the modulation amplitude in a single SP and the (upturned and normalised) kinetics of its overall signal taken at 620nm.

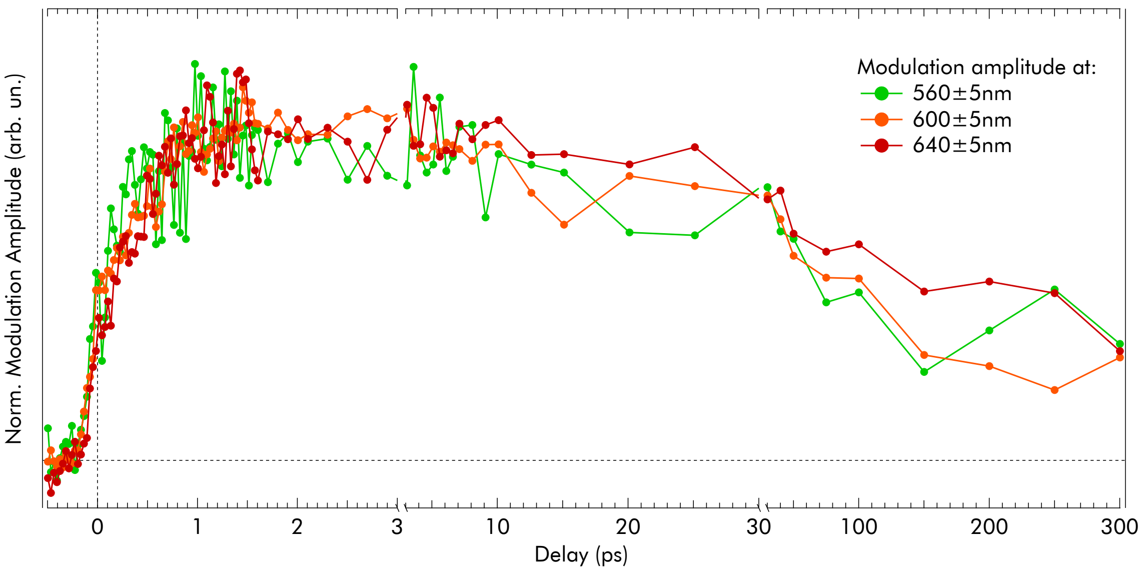

Figure S 13: Time evolution of the modulation amplitude of a single SP evaluated at three different spectral position, highlighting the wavelength-independence of such dynamics.

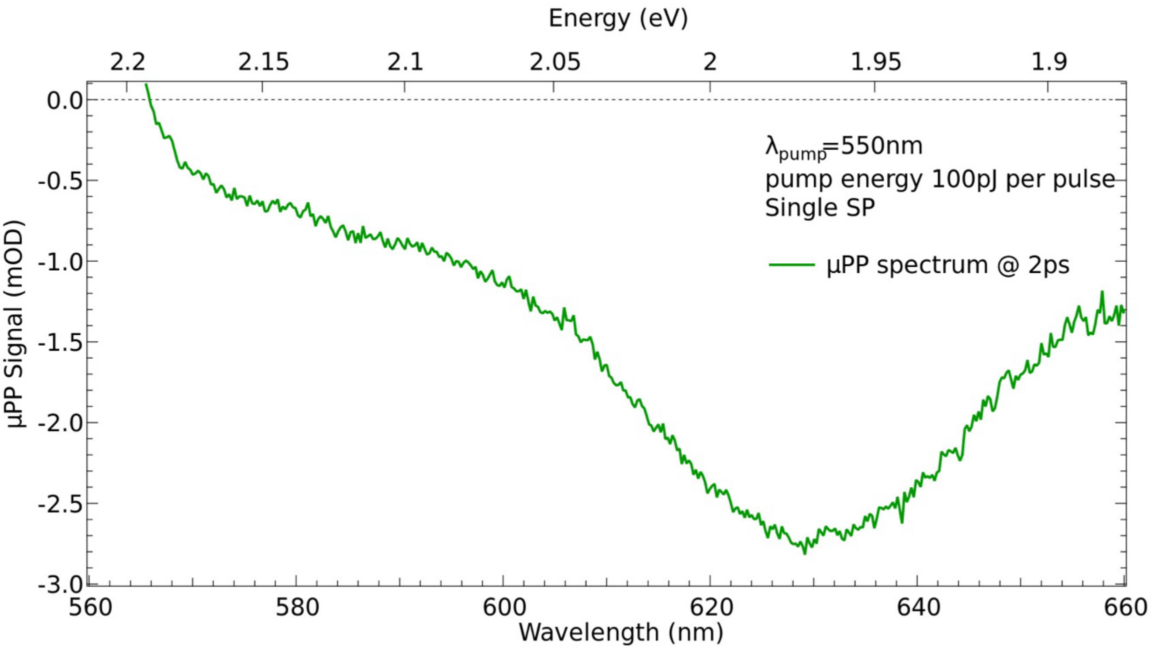

Figure S 14: µPP spectrum of a single SP excited at 550nm, at 2ps delay, highlighting the absence of modulations.

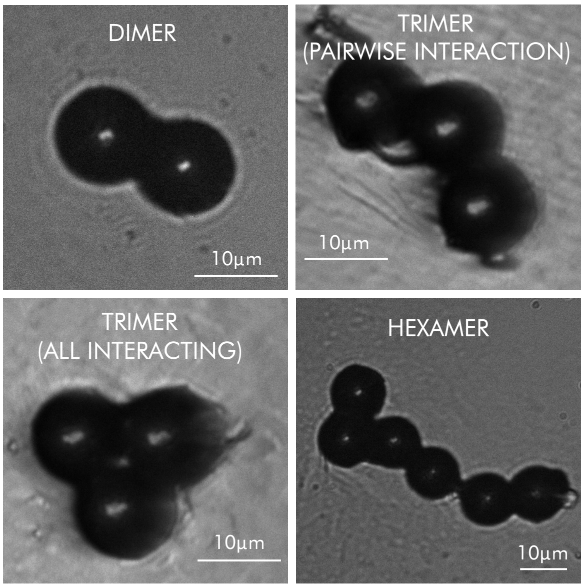

Figure S 15: Micrograph of four distinct multimers, formed by a number of SPs ranging from 2 to 6.

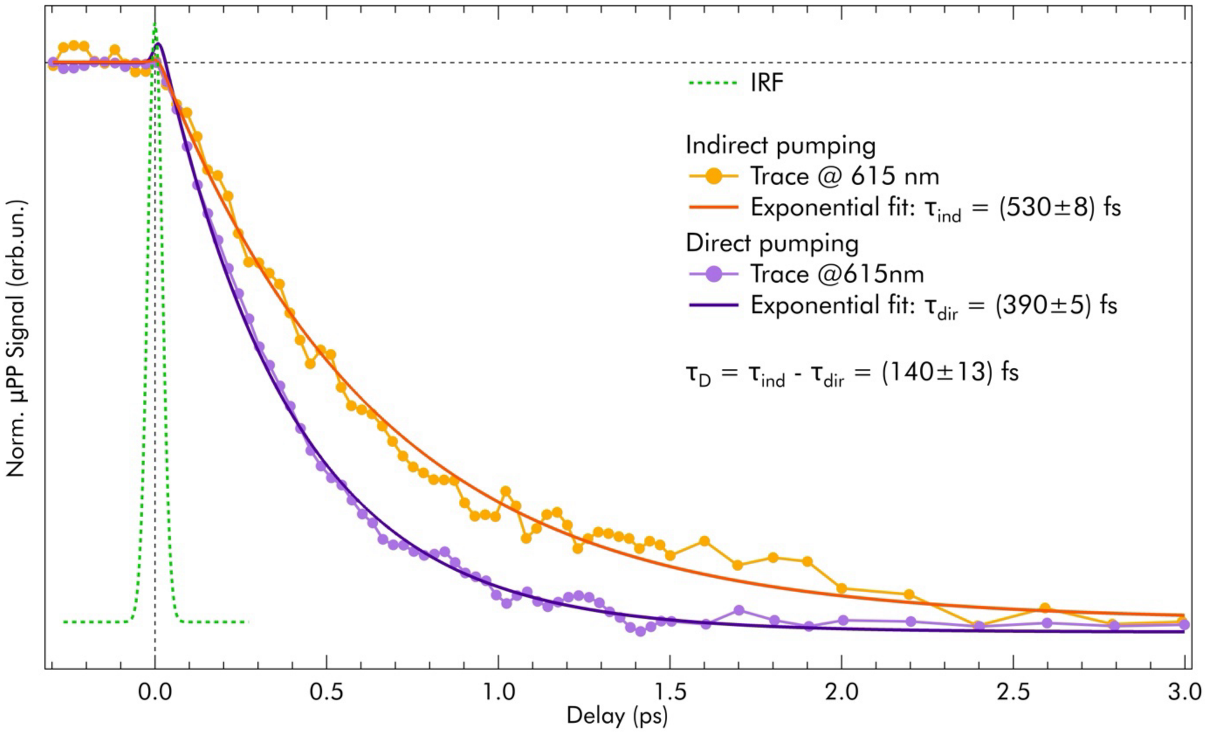

Figure S 16: Early kinetics of the µPP signal of a SP dimer in Indirect and Direct pumping regime fitted with a single exponential convolved with a gaussian instrumental response function. The delay τD in the risetime of the former with respect to that of the latter has been evaluated as the difference between the two rise times. A plot of the instrumental response function is included for clarity.

References

(1) Carbone, L.; Nobile, C.; De Giorgi, M.; Sala, F. Della; Morello, G.; Pompa, P.; Hytch, M.; Snoeck, E.; Fiore, A.; Franchini, I. R.; Nadasan, M.; Silvestre, A. F.; Chiodo, L.; Kudera, S.; Cingolani, R.; Krahne, R.; Manna, L. Synthesis and Micrometer-Scale Assembly of Colloidal CdSe/CdS Nanorods Prepared by a Seeded Growth Approach. *Nano Lett* **2007**, *7* (10), 2942–2950. https://doi.org/10.1021/nl0717661.

(2) Hanifi, D. A.; Bronstein, N. D.; Koscher, B. A.; Nett, Z.; Swabeck, J. K.; Takano, K.; Schwartzberg, A. M.; Maserati, L.; Vandewal, K.; van de Burgt, Y.; Salleo, A.; Alivisatos, A. P. Redefining Near-Unity Luminescence in Quantum Dots with Photothermal Threshold Quantum Yield. *Science (1979)* **2019**, *363* (6432), 1199–1202. https://doi.org/10.1126/science.aat3803.

(3) Marino, E.; van Dongen, S. W.; Neuhaus, S. J.; Li, W.; Keller, A. W.; Kagan, C. R.; Kodger, T. E.; Murray, C. B. Monodisperse Nanocrystal Superparticles through a Source–Sink Emulsion System. *Chemistry of Materials* **2022**, *34* (6), 2779–2789. https://doi.org/10.1021/acs.chemmater.2c00039.

(4) Mann, S. A.; Sciacca, B.; Zhang, Y.; Wang, J.; Kontoleta, E.; Liu, H.; Garnett, E. C. Integrating Sphere Microscopy for Direct Absorption Measurements of Single Nanostructures. *ACS Nano* **2017**, *11* (2), 1412–1418. https://doi.org/10.1021/acsnano.6b06534.

(5) Kovalenko, S. A.; Dobryakov, A. L.; Ruthmann, J.; Ernsting, N. P. Femtosecond Spectroscopy of Condensed Phases with Chirped Supercontinuum Probing. *Phys Rev A (Coll Park)* **1999**, *59* (3), 2369–2384. https://doi.org/10.1103/PhysRevA.59.2369.

(6) de Mello, J. C.; Wittmann, H. F.; Friend, R. H. An Improved Experimental Determination of External Photoluminescence Quantum Efficiency. *Advanced Materials* **1997**, *9* (3), 230–232. https://doi.org/10.1002/adma.19970090308.

(7) Gouesbet, G.; Grehan, G.; Maheu, B. Scattering of a Gaussian Beam by a Mie Scatter Center Using a Bromwich Formalism. *Journal of Optics* **1985**, *16* (2), 83–93. https://doi.org/10.1088/0150-536X/16/2/004.

(8) Bromwich, T. J. Electromagnetic Waves. *Phil.Mag.* **1919**, *38* (223), 143–164.

(9) Davis, L. W. Theory of Electromagnetic Beams. *Phys Rev A (Coll Park)* **1979**, *19* (3), 1177–1179. https://doi.org/10.1103/PhysRevA.19.1177.

(10) Chiasera, A.; Dumeige, Y.; Féron, P.; Ferrari, M.; Jestin, Y.; Nunzi Conti, G.; Pelli, S.; Soria, S.; Righini, G. C. Spherical Whispering-gallery-mode Microresonators. *Laser Photon Rev* **2010**, *4* (3), 457–482. https://doi.org/10.1002/lpor.200910016.

(11) Enríquez, J. Influence of the Thickness on Structural, Optical and Electrical Properties of Chemical Bath Deposited CdS Thin Films. *Solar Energy Materials and Solar Cells* **2003**, *76* (3), 313–322. https://doi.org/10.1016/S0927-0248(02)00283-0.

(12) Lide, D. R. *CRC Handbook of Chemistry and Physics*, 79th ed.; CRCPress: BocaRaton, 1998.

(13) Lisitsa, M. P.; Gudymenko, L. F.; Malinko, V. N.; Terekhova, S. F. Dispersion of the Refractive Indices and Birefringence of $CdS_{x}Se_{1-x}$ Single Crystals. *physica status solidi (b)* **1969**, *31* (1), 389–399. https://doi.org/10.1002/pssb.19690310146.
